# Evaluating the Impact of the COVID-19 Pandemic on Telepharmaceutical Service Effectiveness: Systematic Review and Meta-Analysis

**DOI:** 10.2196/64073

**Published:** 2025-07-02

**Authors:** Puwen Zhang, Mengting Yang, Siyi He, Xiayan Li, Bingchen Lang, Linan Zeng, Lingli Zhang

**Affiliations:** 1 Department of Pharmacy/Evidence-Based Pharmacy Center Children's Medicine Key Laboratory of Sichuan Province West China Second University Hospital, Sichuan University Chengdu China; 2 National Medical Products Administration Key Laboratory for Technical Research on Drug Products In Vitro and in Vivo Correlation Chengdu China; 3 Key Laboratory of Birth Defects and Related Diseases of Women and Children Ministry of Education Chengdu China; 4 West China School of Medicine Sichuan University Chengdu China; 5 West China School of Pharmacy Sichuan University Chengdu China; 6 West China Biomedical Big Data Center West China Hospital Sichuan University Chengdu China; 7 Chinese Evidence-based Medicine Center West China Hospital Sichuan University Chengdu China

**Keywords:** telepharmacy, telepharmaceutical services, pharmacists, COVID-19, meta-analysis, systematic review

## Abstract

**Background:**

Telepharmaceutical services (TPS) led by pharmacists, an emerging telehealth service, improve access to medical services and enable patients to receive specialized services in areas with limited resources. With a lower risk of infection and no restriction of isolation measures, TPS showed great potential during the COVID-19 pandemic. However, whether the effectiveness of TPS changed before and after the outbreak of the COVID-19 pandemic remained unclear.

**Objective:**

This study aimed to evaluate the effectiveness of TPS, compare the effectiveness before and after the outbreak of the COVID-19 pandemic, and explore whether the effectiveness changed over time.

**Methods:**

We searched PubMed, Embase (Ovid), SinoMed, China National Knowledge Infrastructure, Wanfang, and VIP databases for randomized controlled trials that evaluated the effectiveness of TPS. The search covered studies published from inception to October 24, 2023. Eligible studies were conducted before May 5, 2023, when the World Health Organization (WHO) declared the end of the COVID-19 pandemic as a Public Health Emergency of International Concern. We used the random-effect model to pool the results and the Grading of Recommendations Assessment, Development, and Evaluation (GRADE) system to assess the certainty of evidence. To explore whether the effectiveness of TPS changed over time, we applied subgroup analyses (studies conducted before December 31, 2019, and studies conducted after January 1, 2020). Using the independent-sample *z* test, we compared the effectiveness of TPS between the 2 subgroups. When a significant difference arose between them, we conducted a meta-regression analysis to further evaluate the trend of effectiveness over time.

**Results:**

In addition, 40 studies were finally included. Compared with no TPS or usual care (ie, face-to-face pharmaceutical services), TPS probably increased patient medication adherence (risk difference [RD] 0.15, 95% CI 0.09-0.20, moderate certainty), and may reduce the occurrence of adverse events (RD –0.10, 95% CI –0.18 to –0.02, low certainty) and improve the proportion of patients who were satisfied with medication (RD 0.16, 95% CI 0.05-0.26, low certainty). Moderate to high evidence indicated that patients accepting TPS probably achieved superior management of diabetes and hypertension. The effectiveness of TPS was not significantly different before and after the outbreak of the COVID-19 pandemic except for medication adherence (RD 0.12, 95% CI 0.03-0.21, *P*=.007), which also increased over time (coefficient=0.01, 95% CI 0.01-0.02, *P*<.001).

**Conclusions:**

TPS probably improved patient medication adherence and may lead to better satisfaction and the incidence of adverse events. The effectiveness of TPS in general did not change after the outbreak of the COVID-19 pandemic.

**Trial Registration:**

PROSPERO CRD42023487476; https://tinyurl.com/3s47enj6

## Introduction

Telepharmaceutical services (TPS) led by pharmacists were an emerging telehealth service, including pharmaceutical counseling sessions, medication therapy management, drug review and monitoring, clinical consultation, and some other pharmacy services delivered through telecommunications technology [1]. TPS showed potential benefits in the management of chronic disease management, particularly for rural and geographically isolated areas [2-4]. Especially, the COVID-19 pandemic increased the demand of patients for TPS, leading to a surge in use. Patients with potential medical and pharmaceutical problems may be in greater need of professional care after the outbreak of the COVID-19 pandemic than before since those were more susceptible to COVID-19 infection. In addition, patients who accepted regular pharmaceutical services before the outbreak of the COVID-19 pandemic had difficulty in accessing care after the outbreak of the COVID-19 pandemic due to quarantine measures, conducted by countries to isolate the transmission of the virus [5]. Thus, they had to turn to TPS.

Previous systematic reviews related to TPS synthesized results for effective implications. Furthermore, 2 systematic reviews compared the effectiveness of TPS versus no TPS or face-to-face pharmaceutical services, with evidence that TPS appeared to be at least as effective as face-to-face care in the management of diabetes and anticoagulation [6,7]. Two additional systematic reviews after the outbreak of the COVID-19 pandemic supported effective TPS in a qualitative way [8]. However, these previous systematic reviews did not include patient-reported outcomes (ie, health-related quality of life, satisfaction, health behavior, and medication adherence), which were important for assessing how patients were feeling and functioning when evaluating the interventions [9]. More importantly, no evidence compared the difference in TPS before and after the outbreak of the COVID-19 pandemic and analyzed the effectiveness of TPS over time. Compared with TPS before the outbreak of the COVID-19 pandemic, whether TPS addressed the patients’ needs adequately or not after the outbreak remained unclear. This uncertainty has posed a barrier to deciding whether TPS should be improved on a large scale in the future.

We performed a systematic review to evaluate the effectiveness of TPS, compare the effectiveness of TPS before and after the outbreak of the COVID-19 pandemic, and analyze the effectiveness of TPS with time passing. This study included the most comprehensive and latest studies about TPS, which may provide useful evidence of TPS and policy making in the post–COVID-19 pandemic era.

## Methods

We registered this study in PROSPERO (International Prospective Register of Systematic Reviews; CRD42023487476). We revised the protocol after registration (Multimedia Appendix 1). We reported this study according to the PRISMA (Preferred Reporting Items for Systematic Reviews and Meta-Analyses) statement (Multimedia Appendix 2) [10].

### Search Strategy and Selection Criteria

We searched PubMed, Embase (Ovid), SinoMed, China National Knowledge Infrastructure, Wanfang, and VIP databases from inception to October 24, 2023 (Multimedia Appendix 3). In addition, 2 reviewers independently screened the studies based on titles and abstracts. Reviewers had a discussion in case of disagreement to reach a consensus with the guidance of the third reviewer.

We set the inclusion criteria based on Population, Intervention, Comparison, and Outcome principles [11]. Eligible studies were randomized controlled trials relevant to TPS led by pharmacists. The authors defined the comparison between TPS and no TPS or usual care (ie, face-to-face pharmaceutical services). To compare the effectiveness of TPS before and after the outbreak of the COVID-19 pandemic through prespecified subgroup analyses, we included studies conducted before May 5, 2023, when the World Health Organization (WHO) declared COVID-19 was no longer a Public Health Emergency of International Concern [12]. To ensure comparability, we only selected studies from countries with the same income level before and after the outbreak. We excluded studies related to other telehealth services, even if they involved pharmaceutical care, or if pharmacists were only involved in supporting a research role (ie, recruitment). We excluded studies without full-text or published in languages other than English and Chinese. We also excluded the studies with a significant difference in the baseline for outcomes of interest between the comparison (*P*≤.05).

### Outcomes of Interest

The primary outcomes of interest were medication adherence, medication satisfaction, and adverse events. We focused on the multiple measures of medication adherence, including pharmacy-based data (eg, the proportion of days covered), patient self-report measures (eg, measured by questionnaires, scales, or instruments), and reports from health care professionals or carers. Similarly, satisfaction, another patient-reported outcome, was evaluated in multiple forms (eg, proportion of satisfaction for patients and score of satisfaction with different scales). We also assessed other disease-specific measures (eg, hemoglobin A_1c_ [HbA_1c_], systolic blood pressure [SBP], diastolic blood pressure [DBP], and international normalized ratio [INR]). We were interested in endpoints at the end of the follow-up, not changing indexes during the follow-up.

### Data Extraction

In addition, 2 reviewers (PZ and XL) independently extracted the following information: regions and income level of countries, year of study conduction, type of intervention, characteristics of participants, and outcomes of interest with data. Country regions and income levels were classified according to the World Bank (2022-2023) [13].

### Risk-of-Bias Assessment and Certainty of Evidence

Using the Cochrane Risk of Bias (RoB) tool, 2 reviewers (PZ and XL) assessed the RoB of the methodological quality of eligible individual studies [14]. We summarized the overall RoB for each study with low, moderate, or high risk (Multimedia Appendix 4). Applying the Grading of Recommendations Assessment, Development, and Evaluation (GRADE) system, 2 reviewers (PZ and XL) rated the certainty of a body of evidence regarding the specific outcome in the meta-analysis independently (Multimedia Appendix 5) [15]. We rated the certainty of evidence in GRADE based on whether TPS had a true effect. Certainty reflected our confidence that the observed effects differed from the null [16,17]. We used a null effect as the threshold in absolute effect size and assessed the certainty based on absolute measures (eg, risk difference [RD] and mean difference) rather than relative measures (eg, risk ratio). When facing disagreement, the 2 reviewers reached a consensus by discussing with the third reviewer.

### Data Analysis

For dichotomous outcomes (eg, medication adherence or patient satisfaction), we calculated risk ratio and RD with 95% CI values to estimate the combined effect size associated with TPS.

For continuous outcomes (eg, score of satisfaction and medication adherence with different scales), based on the extracted means and SDs between the comparisons, we calculated mean difference with 95% CI values. If SDs were unavailable, we calculated values by converting SEs or CIs, as informed in Chapter 7.7.3.2 of the Cochrane Handbook [18].

We used a random-effect model to pool effect estimates [19]. Pooling effect estimates of medication adherence and satisfaction were difficult because of different measures. To address this problem, we reported data in the original form defined by the authors (dichotomous and continuous data). To simplify the pooled process of continuous data, we transferred the total scale to 100 points. Using Funnel plots and Egger test (≥10 included studies), we assessed potential publication bias [20]. We also made sensitivity analyses to assess the robustness of the synthesized results.

### Subgroup Analyses

#### Prespecified Subgroup Analysis: TPS Before and After the Outbreak of COVID-19

According to the year of conduction, we categorized the studies into 2 subgroups: before and after the outbreak of the COVID-19 pandemic. We defined “before the outbreak of the COVID-19 pandemic” as studies conducted before December 31, 2019—the date of WHO’s first official report about the same cluster of cases of “pneumonia of unknown cause” in Wuhan [21-23]—and “after the outbreak of COVID-19” as studies conducted after January 1, 2020.

We tested a priori hypothesis that effect sizes in the “after the outbreak of the COVID-19 pandemic” subgroup would be different statistically from the “before the outbreak” subgroup because of the rapid advancement of telehealth during the COVID-19 pandemic. Using the independent-sample *z* test, we explored the difference in TPS before and after the outbreak of the COVID-19 pandemic [24]. For each outcome, we calculated the absolute effect size difference (RD or mean difference) and its corresponding SE between subgroups (Multimedia Appendix 6). We then computed the *z* value and *P* value. If the subgroup difference was significant (*P≤*.05), we further performed a univariate meta-regression analysis using the year in which the included studies were conducted as the independent variable to explore potential temporal trends.

#### Post Hoc Subgroup Analysis: TPS by Intervention Types and Geographic Regions

For the primary outcomes, we conducted 2 additional subgroup analyses. First, we categorized TPS by intervention type into “TPS by pharmacists” and “TPS by pharmacists with a telemonitoring device” to assess their impact on effectiveness. Second, we performed regional subgroup analyses to examine potential geographical variations. We conducted all the analyses in RStudio (version 4.3.3). The *P* values were 2-sided, with an α level of .05 considered significant.

### Ethical Considerations

This study is a systematic review and meta-analysis based on previously published trials. It does not involve any individual patient data collection or new clinical interventions. Therefore, ethical approval was not required.

## Results

### Overview

**Figure 1:**
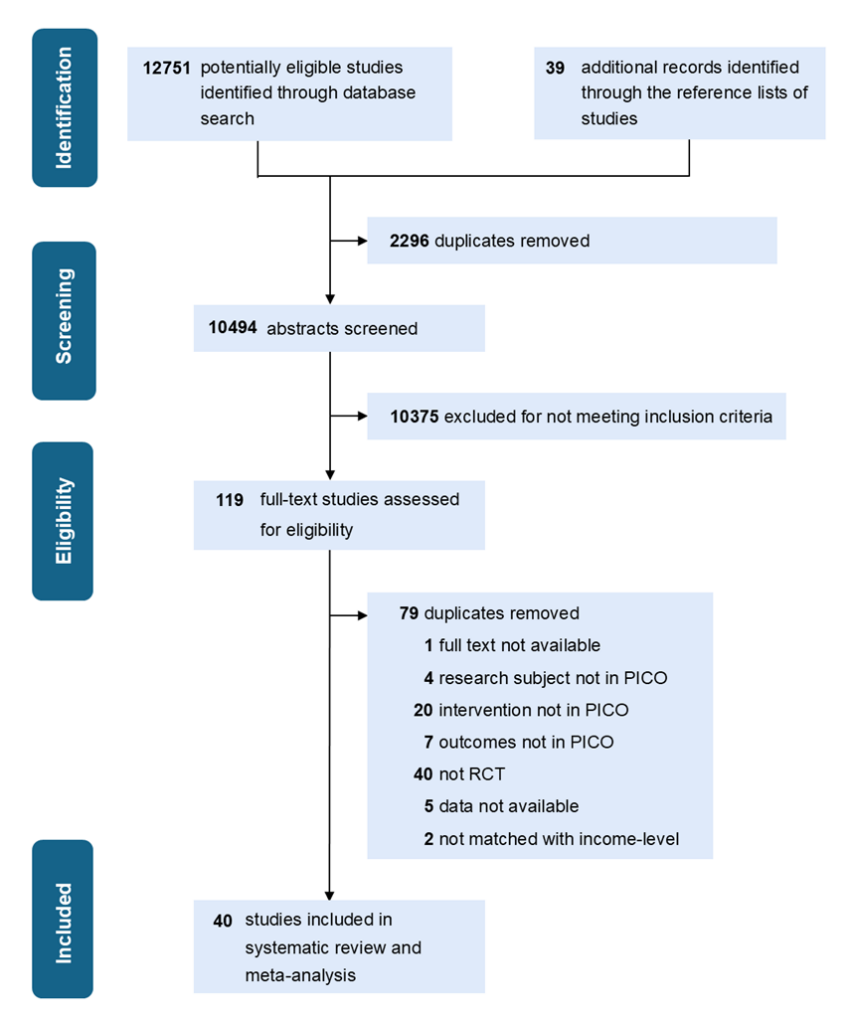
Literature search and selection. PICO: Population, Intervention, Comparison, and Outcome; RCT: randomized controlled trial.

**Figure 2:**
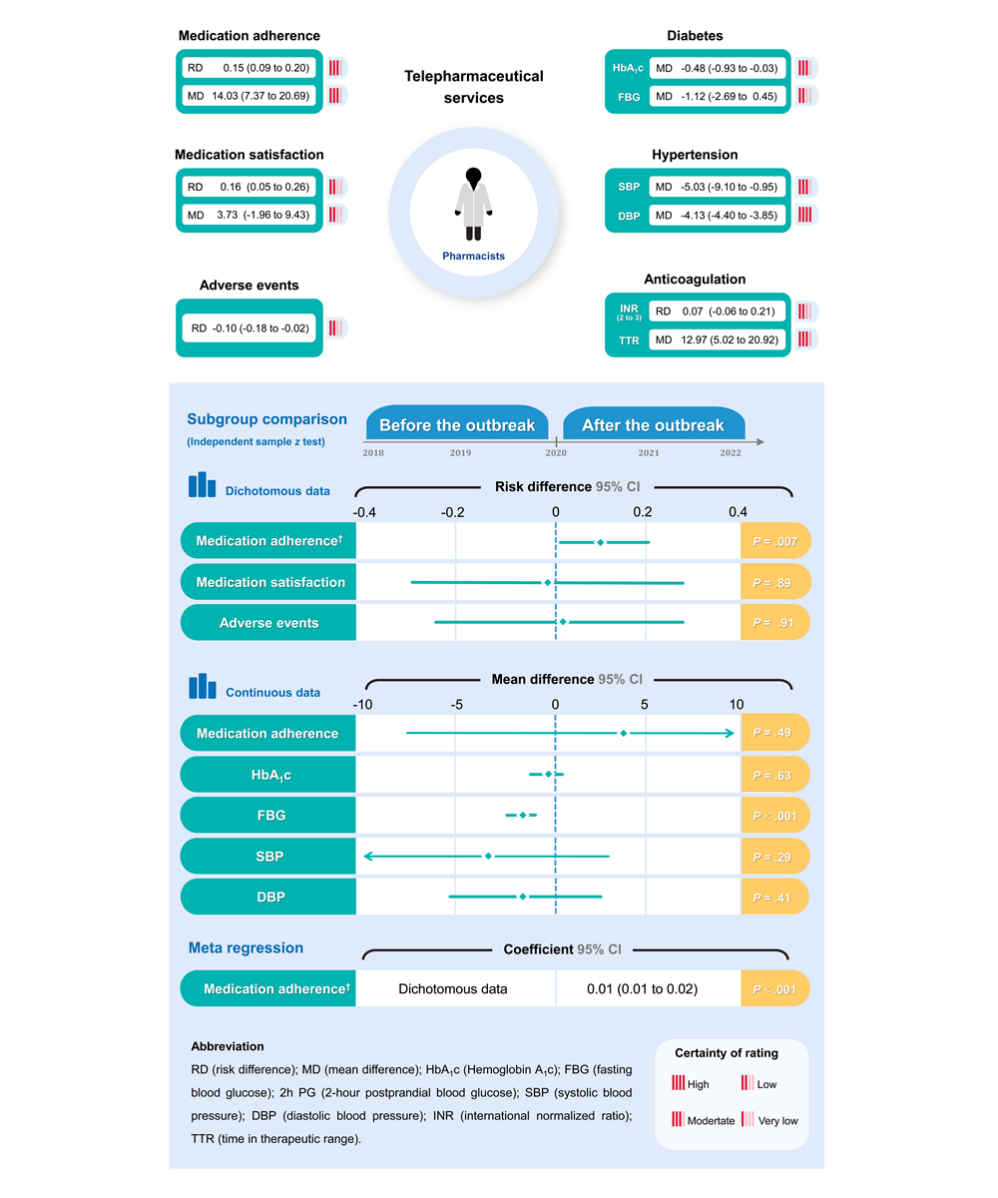
Summary of results.

The literature search in databases yielded 12,751 studies, and citation searching yielded another 39 studies. A total of 40 studies that met the eligibility criteria were finally included (Figures 1 and 2 and Multimedia Appendix 7) [25-64].

### Study Characteristics and RoB Assessment

**Figure 3:**
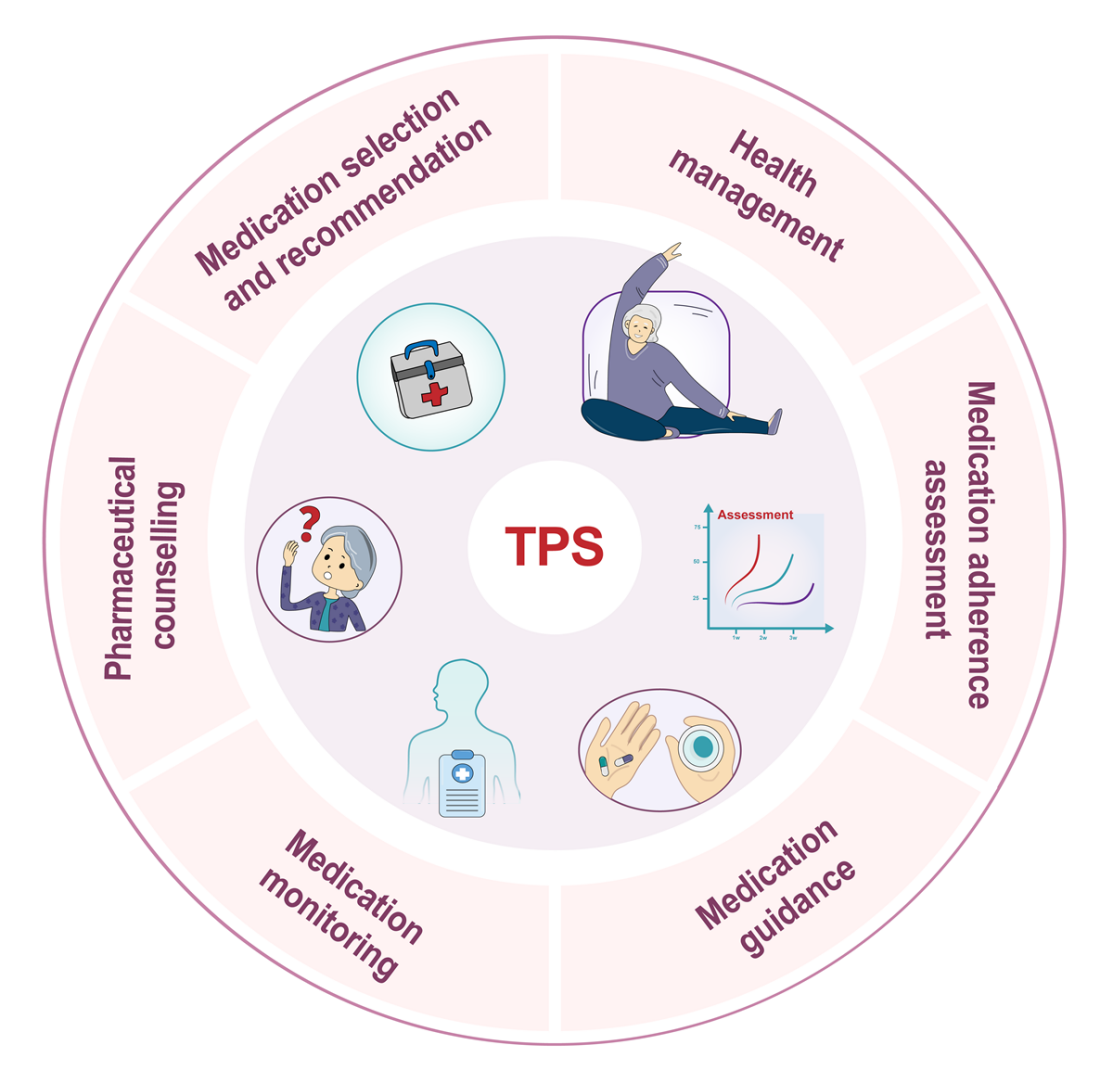
Components and characteristics of TPS. TPS: telepharmaceutical services.

A total of 27 out of 40 (67.5%) studies were conducted before the outbreak of the COVID-19 pandemic [25-51] and 13 out of 40 (32.5%) studies after the outbreak (Multimedia Appendix 8) [52-64]. All 40 studies reported overlapping and various formats of TPS, including medication selection and recommendation, pharmaceutical counseling, medication monitoring, medication adherence assessments, medication guidance, and health management (Figure 3 and Multimedia Appendix 9) [25-64]. A total of 34 out of 40 (85%) studies were categorized as TPS by pharmacists [25-27,32,34-41,43-64], while the other 6 out of 40 (15%) studies involved TPS by pharmacists assisted by a telemonitoring device focused on home blood pressure monitoring (Multimedia Appendix 9) [28-31,33,42]. A total of 23 out of 40 (57.5%) studies were from East Asia and Pacific [37-39,41,43-50,52-57,59-63], 13 out of 40 (32.5%) studies from North America [25,26,28-34,36,40,42,51], 2 out of 40 (5%) studies from Europe and Central Asia [27,35], 2 out of 40 (5%) studies from Middle East and North Africa [58,64], and none from Latin America and Caribbean, South Asia, or Sub-Saharan Africa.

A total of 11 out of 40 (27.5%) studies identified as high RoB [26,45,49,50,54,56,58-62], 21 out of 40 (52.5%) studies as moderate [25,27,29,33-35,38-40,42-44,46-48,52,53,55,57,63,64] and 8 out of 40 (20.0%) studies as low (Multimedia Appendix 4) [28,30-32,36,37,41,51]. The major concern is performance bias. None of the 40 studies blinded participants, and only 5 (12.5%) studies blinded outcome assessors [28,29,36,40,46]. Outcomes with a larger sample size did not find publication bias in the proportion of medication adherence (*P*=.29) or adverse events (*P*=.20) with the Egger test (Multimedia Appendix 10).

### Effectiveness of TPS

#### Medication Adherence

TPS probably increased the proportion of patients who achieved medication adherence (RD 0.15, 95% CI 0.09-0.20, moderate certainty) and the mean medication adherence score (mean difference 14.03, 95% CI 7.37-20.69 on a 0-100 scale, moderate certainty) versus no TPS or usual care (Multimedia Appendix 11).

#### Medication Satisfaction

The proportion of patients who were satisfied with medication in the TPS group may be higher than that in the control group (RD 0.16, 95% CI 0.05-0.26, low certainty), while the effect of the mean satisfaction score was nonsignificant (mean difference 3.73, 95% CI –1.96 to 9.43 on a 0-100 scale, low certainty; Multimedia Appendix 11).

#### Adverse Events

Compared with no TPS or usual care, TPS may lead to fewer adverse events (RD –0.10, 95% CI –0.18 to –0.02, low certainty; Multimedia Appendix 11).

### Disease-Specific Measures

#### Diabetes

Compared with the control group, patients accepting TPS probably achieved greater glucose management of HbA_1c_ (mean difference –0.48, 95% CI –0.93 to –0.03, moderate certainty) and 2-hour postprandial blood glucose (mean difference –2.77, 95% CI –3.47 to –2.07, moderate certainty). TPS had a nonsignificant statistical reduction in fasting blood glucose (FBG) versus no TPS or usual care (mean difference –1.12, 95% CI –2.69 to 0.45, low certainty; Multimedia Appendix 11).

#### Hypertension

Compared with no TPS or usual care, TPS probably had a better blood pressure control of SBP (mean difference –5.03, 95% CI –9.10 to –0.95, moderate certainty) and DBP (mean difference –4.13, 95% CI –4.40 to –3.85, high certainty), achieving minimally important difference (MID; MID_SBP_=3 mm Hg, MID_DBP_=2 mm Hg; Multimedia Appendix 11) [65].

#### Anticoagulation

Current guidelines recommend that warfarin adjusted to a value of INR of 2-3 is most advantageous for patients after trading off the benefits and risks [66]. TPS may increase the proportion of INR of 2-3 (RD 0.07, 95% CI –0.06 to 0.21, low certainty), despite no statistically significant improvement. In addition, moderate-certainty evidence indicated TPS probably increased time within the therapeutic range (TTR; mean difference 12.97, 95% CI 5.02-20.92, moderate certainty; Multimedia Appendix 11).

#### Other Diseases

Patients with stroke accepting TPS may result in a lower recurrence (mean difference –0.20, 95% CI –0.38 to –0.02, low certainty) and a higher Barthel index score (mean difference 14.75, 95% CI 8.78-20.72, low certainty) versus no TPS or usual care. Regarding patients with cancer, the evidence is very uncertain about the effect of TPS on pain (mean difference –2.39, 95% CI –2.56 to –2.22, very low certainty). In terms of respiratory diseases, TPS probably improved peak expiratory flow (mean difference 88.66, 95% CI 60.03-117.29, moderate certainty) and respiratory function (mean difference –0.69, 95% CI –0.72 to –0.66, moderate certainty), and might improve forced expiratory volume in one second (mean difference 8.51, 95% CI 6.77-10.25, low certainty; Multimedia Appendix 11).

### Subgroup Analyses

#### Comparison of the Effectiveness of TPS Before and After the Outbreak of the COVID-19 Pandemic

**Figure 4:**
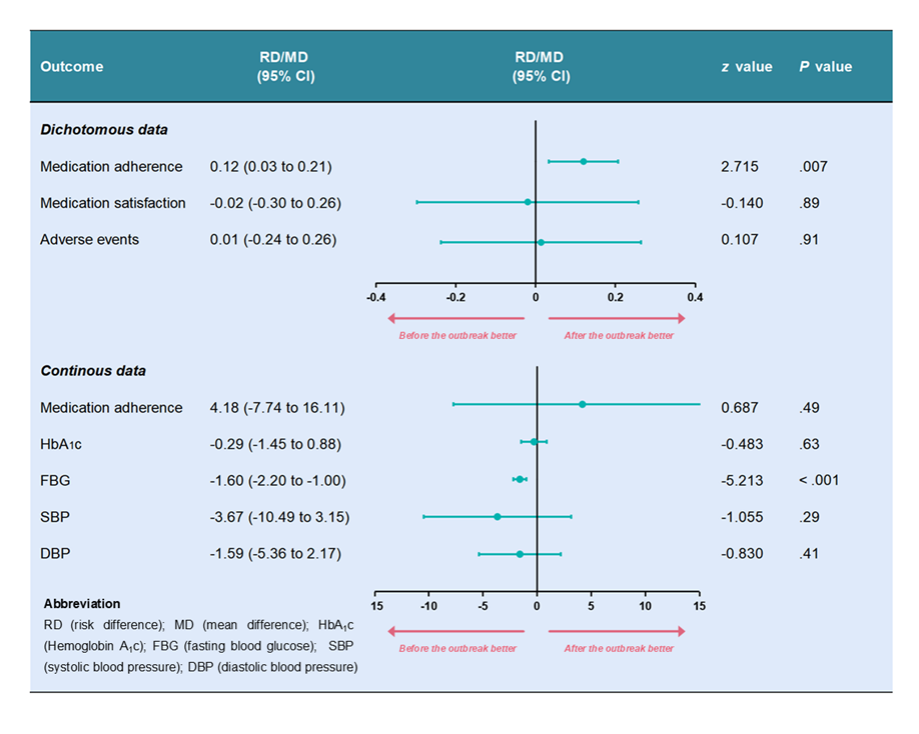
Subgroup analysis with independent-sample z test.

An independent-sample *z* test showed significant differences between subgroups (before and after the outbreak of the COVID-19 pandemic) for the proportion of patients who achieved medication adherence (*P*=.007) and FBG (*P*<.001; Figure 4). However, only the proportion of patients who achieved medication adherence displayed a positive subgroup difference (RD 0.12, 95% CI 0.03-0.21, *P*=.007), suggesting a potential improvement in medication adherence after the outbreak of the COVID-19 pandemic.

Meta-regression further revealed a positive association between medication adherence and conducted year of study (coefficient=0.01, 95% CI 0.01-0.02, *P*<.001), indicating a progressive improvement in TPS effectiveness over time (Multimedia Appendix 12). We did not perform a meta-regression for FBG because only 2 studies were included.

#### Comparison of the Effectiveness of TPS by Intervention Types and Geographic Regions

The intervention-based subgroup analysis showed that the effectiveness of TPS by pharmacists with telemonitoring was similar to that of TPS by pharmacists in point estimates (Multimedia Appendix 13). However, the combined intervention had a smaller sample size, which likely led to the lack of statistical significance, as the confidence intervals crossed the null effect. In the regions-based subgroup analysis, TPS may show potential effectiveness across different regions overall, although the effect size might vary (Multimedia Appendix 14).

### Sensitivity Analyses

We conducted sensitivity analyses to assess the robustness of the results regarding the effectiveness of TPS. Specifically, we recalculated the pooled effect estimates by removing each included study. Detailed results of the sensitivity analyses are provided in Multimedia Appendix 15.

## Discussion

### Principal Findings

This systematic review showed that TPS may result in a benefit in patient medication adherence, the proportion of satisfaction, and adverse events. For disease-specific measures, TPS also displayed larger point estimates in the control of diabetes, hypertension, anticoagulation, stroke, and respiratory diseases. The effect of TPS on cancer pain was uncertain due to only one included study though the effect size was attenuated versus no TPS or usual care. Subgroup analysis before and after the outbreak of the COVID-19 pandemic suggested no statistical difference in the outcomes of interest, except the proportion of medication adherence and FBG. Meta-regression analysis verified a positive association between the proportion of medication adherence and time passing.

### Comparison With Previous Work

The studies by Moschonis et al [67] and Siopis et al [68] evaluated the effectiveness of SMS, smartphone app, and website interventions on adults with diabetes and hypertension. Our study and that of and Moschonis et al [67] showed that TPS could decrease HbA_1c_ (mean difference –0.30% and –0.48%) versus usual care. For hypertension, our study and that of Siopis et al [68] similarly displayed that TPS could achieve better management of SBP (mean difference –3.62 and –5.03 mm Hg) and DBP (mean difference –2.45 and –4.13 mm Hg). The point estimates in our study seemed to be larger than theirs. We might conclude that pharmacists with professional knowledge may exert a more significant function than simple digital health intervention in the management of chronic diseases.

Our study showed that TPS may increase the proportion of patients who were satisfied with their medication but did not increase the mean score of patients’ medication satisfaction statistically. The point estimate in the mean score of satisfaction in the TPS group was still higher than that in the control group though no statistical difference. For one thing, only 2 studies with small sample sizes were included in the score of satisfaction. Moreover, the attitude of patients to TPS may not be consistent actually. Previous qualitative interviews might account for this phenomenon. Some patients with positive feelings for TPS may deem TPS as time or cost-saving, flexible, and accessible care while others with negative feelings may think TPS lacked adequate clinical assessment and threatened their confidentiality [69-71].

### Reasons for the Limited Improvement of TPS After the Outbreak of COVID-19

Telehealth services, vital measures in response to the pandemic, have expanded substantially during the COVID-19 pandemic [72]. TPS, one form of telehealth services, made a rapid advancement as well. Nevertheless, the benefits of TPS, in general, seemed independent whether they occurred before or after the outbreak of the COVID-19 pandemic. Several reasons may explain this limited improvement.

One key reason could be the challenges TPS faced due to the pandemic. In contrast with TPS before the outbreak of COVID-19, the number of patients accepting TPS was increasing rapidly due to quarantine after the outbreak. This surge in demand might have overwhelmed the system, making it difficult to maintain quality service [71]. Furthermore, many pharmacists said that they were not ready to deal with the disasters when facing the COVID-19 pandemic [69]. Although the pandemic sparked increased attention toward TPS, the anticipated improvement in its effectiveness did not materialize as expected. The rapid shift to telehealth, while essential, may have outpaced the adaptation of health care providers.

### Strengths and Limitations

Our study had the following strengths. First, to our knowledge, this systematic review provided the most comprehensive summary of the available evidence on the effectiveness of TPS up to now. Different from previous systematic reviews [6-8,73-75], we evaluated all important outcomes for patients, including clinical outcomes and patient-reported outcomes. Second, to explore the impact of COVID-19 on TPS, we compared the effect size of TPS before and after the outbreak of the COVID-19 pandemic. Third, we provided the certainty of evidence using the GRADE approach.

This systematic review also had some limitations. First, the generalizability to different income contexts may be limited. Studies included were from higher-income countries with a lower prevalence of poverty, which may not fully represent the lower-income countries. Certainly, we acknowledged implementation of TPS in lower-income countries was challenging due to limited resources. This may account for why the studies came from higher-income countries. Meanwhile, the practice of TPS in lower-income countries warrants examination. Second, some studies displayed relatively low quality attributed to the unclear descriptions of methodology. More high-quality studies will be also needed in the future. Third, heterogeneity between studies was not ideal. An inherent source of heterogeneity may be TPS itself. Because constrained by culture and awareness, TPS across the included studies have different components (Multimedia Appendix 9). Patient characteristics also contributed, including their attitude toward TPS, disease severity, and digital literacy. In addition, pharmacists’ capabilities of conducting TPS varied and provided different extents of attention for patients, which may have further introduced heterogeneity. Fourth, we did not assess the impact of pandemic-related policies (eg, lockdowns and restrictions of entry into public localities) when comparing the effectiveness of TPS before and after the outbreak of COVID-19. Different countries altered the prevention and control policies aligned with the condition of the pandemic after the outbreak of COVID-19, which might also influence health care practices (eg, increased attention to patients) and patient behavior regarding TPS (eg, increased demand for pharmaceutical counseling). Fifth, we did not evaluate the effectiveness of TPS in the post–COVID-19 era, when most countries lifted quarantine restrictions. Our search strategy was set on October 24, 2023, and we have not retrieved eligible studies conducted in the post–COVID-19 era.

### Implications for Future Research

Our systematic review highlighted several areas for future research. First, more original studies are needed to examine the impact of the COVID-19 outbreak and related policies, such as controlled before-after studies and interrupted time series studies. Second, systematic reviews that incorporated these original studies are also needed to provide a comprehensive analysis of the impact. Third, future studies could include research conducted in the post–COVID-19 era. These studies could categorize the timeline into 3 periods to update our findings and further assess the effectiveness of TPS over time.

### Conclusion

This systematic review suggested that TPS probably enhanced patient medication adherence, might have improved medication satisfaction, and might reduce the incidence of adverse events. However, no positive significant changes except for the proportion of patients who achieved medication adherence in the comparison of before and after the outbreak of the COVID-19 pandemic. The effectiveness of TPS may vary depending on context-specific factors, and further research is needed to better understand these variations. In the long term, performing TPS may be an important approach for lower costs and resources in the long run. Therefore, our findings sound a call to the relevant stakeholders, emphasizing the improvement of TPS in the post–COVID-19 era is still needed to better address patients’ requirements.

## Appendix

Multimedia Appendix 1 Amendments to the protocol.

Multimedia Appendix 2 PRISMA (Preferred Reporting Items for Systematic Reviews and Meta-Analyses) checklist.

Multimedia Appendix 3 Search strategies.

Multimedia Appendix 4 Risk of bias for included studies.

Multimedia Appendix 5 GRADE (Grading of Recommendations, Assessment, Development, and Evaluations) for evidence.

Multimedia Appendix 6 The independent sample z test strategies and methods.

Multimedia Appendix 7 Summary of findings.

Multimedia Appendix 8 Characteristics of included studies.

Multimedia Appendix 9 Classification and characteristic of telepharmaceutical services.

Multimedia Appendix 10 Funnel plots.

Multimedia Appendix 11 Forest plots for meta-analysis.

Multimedia Appendix 12 Bubble plots for meta-regression.

Multimedia Appendix 13 Subgroup analysis for telepharmaceutical services by intervention type.

Multimedia Appendix 14 Subgroup analysis for different regions of telepharmaceutical services.

Multimedia Appendix 15 Sensitivity analyses.

## Abbreviations

DBP: diastolic blood pressure
FBG: fasting blood glucose
GRADE: Grading of Recommendations, Assessment, Development, and Evaluations
HbA1c: hemoglobin A1c
INR: international normalized ratio
MID: minimally important difference
PRISMA: Preferred Reporting Items for Systematic Reviews and Meta-Analyses
PROSPERO: International Prospective Register of Systematic Reviews
RD: risk difference
RoB: risk of bias
SBP: systolic blood pressure
TPS: telepharmaceutical services
TTR: time within the therapeutic range
WHO: World Health Organization
