# Supplementary material for: Evaluating the Impact of the COVID-19 Pandemic on Telepharmaceutical Service Effectiveness: Systematic Review and Meta-Analysis
Source: J Med Internet Res. 2025 Jul 2;27:e64073. doi: 10.2196/64073 (PMC12268221; doi:10.2196/64073)
Supplement: Multimedia Appendix 1 [file jmir_v27i1e64073_app1.pdf]

Multimedia Appendix 1: Amendments to protocol

We registered this systematic review in the PROSPERO (CRD42023487476) in advance and initiated in October 2023. Several amendments or clarifications were made. First, we only included randomized controlled trials contributing to quantitative systematic review substitute of mixed-methods systematic review. For one thing, we want to include studies with higher certainty evidence. For the other thing, we do not find an appropriate association to incorporate the qualitative and quantitative studies to stress some specific themes. Second, we added some randomized controlled trials conducted before the outbreak of the COVID-19 pandemic. That's because we wanted to detect the impact of COVID-19 on telepharmacetical service (TPS). Adding these studies, this systematic review could provide comprehensive evidence related to TPS.
