# Supplementary material for: Evaluating the Impact of the COVID-19 Pandemic on Telepharmaceutical Service Effectiveness: Systematic Review and Meta-Analysis
Source: J Med Internet Res. 2025 Jul 2;27:e64073. doi: 10.2196/64073 (PMC12268221; doi:10.2196/64073)
Supplement: Multimedia Appendix 3 [file jmir_v27i1e64073_app3.pdf]

Multimedia Appendix 3: Search strategies

3.1 PubMed (English)

((("Pharmaceutical Services, Online"[Mesh]) OR (((((("internet pharmac*"[Title/Abstract]) OR ("online pharmac*"[Title/Abstract])) OR ("digital pharmac*"[Title/Abstract])) OR ("telepharmac*"[Title/Abstract])) OR ("tele-pharmac*"[Title/Abstract])) OR ("e-pharmacy"[Title/Abstract]))) OR (((internet[Title/Abstract]) OR (online[Title/Abstract]) OR (digital[Title/Abstract]) OR (tele[Title/Abstract])) AND ("Pharmaceutical Services"[Mesh]))) NOT (student*[Title/Abstract] OR undergraduate*[Title/Abstract] OR postgraduate*[Title/Abstract])

3.2 Embase (Ovid, English)

1	pharmaceutical service.m_titl.
2	internet.m_titl.
3	online.m_titl.
4	pharmaceutical care.m_titl.
5	"internet pharmac* ".m_titl.
6	"online pharmac* ".m_titl.
7	"digital pharmac* ".m_titl.
8	"telepharmac* ".m_titl.
9	e-pharmacy.m_titl.
10	"tele-pharmac* ".m_titl.
11	2 or 3
12	1 and 11
13	4 and 11
14	exp online pharmacy/
15	5 or 6 or 7 or 8 or 9 or 10 or 12 or 13 or 14

3.3 China National Knowledge Infrastructure (Chinese)

SU=('互联网' + '线上' + '远程' + '网上') * ('药学服务' + '药事' + '药品' + '药物' + '处方' + '用药交代' + '用药咨询' + '用药教育' + '药物重整' + '药学门诊') NOT SU=('教学' + '课程')

3.4 Wanfang (Chinese)

题名或关键词:((("互联网" or "线上" or "远程" or "网上") and ("药学服务" or "药事" or "药品" or "药物" or "处方" or "用药交代" or "用药咨询" or " 用药教育" or "药物重整" or "药学门诊")) not ("教学" or "课程"))

3.5 Sinomed (Chinese)

1	"药学服务"[不加权:扩展]
2	"互联网"[摘要:智能] OR "网上"[摘要:智能] OR "线上"[摘要:智能] OR "远程"[摘要:智能] OR "网络"[摘要:智能]
3	(#2) AND (#1)

3.6 VIP (Chinese)

(M=((互联网 OR 线上 OR 远程 OR 网上) AND (药学服务 OR 药事 OR 药品 OR 药物 OR 处方 OR 用药交代 OR 用药咨询 OR 用药教育 OR 药物重整 OR 药学门诊))) NOT M=(教学 OR 课程)
