# Supplementary material for: Evaluating the Impact of the COVID-19 Pandemic on Telepharmaceutical Service Effectiveness: Systematic Review and Meta-Analysis
Source: J Med Internet Res. 2025 Jul 2;27:e64073. doi: 10.2196/64073 (PMC12268221; doi:10.2196/64073)

Multimedia Appendix 4: Risk of bias for included studies

4.1 Risk of bias summary

Study	Random sequence generation	Allocation concealment	Blinding of participants and personnel	Blinding of outcome assessment	Incomplete outcome data	Selective reporting	Other sources of bias	Overall rating (High/Moderate /Low)
Alsabbagh MW 2012	Low	Low	High	Unclear	Low	Low	Unclear	Low
Bynum A 2001	Low	Unclear	High	Unclear	Low	Low	Unclear	Moderate
Chen XQ 2022	Low	Unclear	High	Unclear	Low	Unclear	Unclear	Moderate
Chen Y 2017	Low	Unclear	High	Unclear	Low	Low	Unclear	Moderate
Choudhry KN 2018	Low	Low	High	Low	Low	Low	Unclear	Low
Elliott RA 2008	Unclear	Low	High	Unclear	Low	Low	Unclear	Moderate
Feng H 2021	Unclear	Unclear	High	Unclear	Low	Unclear	Unclear	High
Green BB 2008	Low	Low	High	Low	Low	Low	Unclear	Low
Ibrahim OM 2022	Low	High	High	Unclear	Low	Low	Unclear	High
Jiang H 2022	Low	Unclear	High	Unclear	Low	Unclear	Unclear	Moderate
Jin X 2021	Unclear	Unclear	High	Unclear	Low	Unclear	Unclear	High
Khan YH 2022	Low	Unclear	High	Unclear	Low	Unclear	Unclear	Moderate
Lauffenburger JC 2019	Low	Unclear	High	Low	Low	Low	Unclear	Moderate
Li JY 2022	Low	Unclear	High	Unclear	Low	Unclear	Unclear	Moderate
Liao QQ 2023	Unclear	Unclear	High	Unclear	Low	Unclear	Unclear	High

Liu H 2022	Unclear	Unclear	High	Unclear	Low	Unclear	Unclear	High
Liu Y 2022	Low	High	High	Unclear	Low	Unclear	Unclear	High
Lu Y 2017	Low	Unclear	High	Unclear	Low	Low	Unclear	Moderate
Lu ZW 2021	Low	Unclear	High	Low	Low	Low	Unclear	Moderate
Lyons I 2016	Low	Unclear	High	Unclear	Low	Low	Unclear	Moderate
Magid DJ 2011	Low	Low	High	Unclear	Low	Low	Unclear	Low
Magid DJ 2013	Low	Low	High	Unclear	Low	Low	Unclear	Low
Margolis KL 2013	Unclear	Low	High	Unclear	Low	Low	Unclear	Moderate
Margolis KL 2022	Low	Unclear	High	Unclear	Low	Unclear	Unclear	Moderate
Peasah SK 2020	Low	Unclear	High	Unclear	Low	Unclear	High	Low
Ralston JD 2014	Unclear	Low	High	Low	Low	Low	Unclear	Moderate
Shi NN 2021	Low	Low	High	Unclear	Low	Unclear	Unclear	Low
Staresinic AG 2006	Unclear	Unclear	High	Unclear	Low	Low	Unclear	High
Sudas Na Ayutthaya N 2018	Low	Low	High	Unclear	Low	Low	Unclear	Low
Wan JW 2022	Low	Unclear	High	Unclear	Low	Unclear	Unclear	Moderate
Wang ZM(1) 2023	Low	Unclear	High	Unclear	Low	Unclear	High	High
Wang ZM(2) 2023	Low	Unclear	High	Unclear	Low	Unclear	Unclear	Moderate
Xu JY 2023	Low	Unclear	High	Unclear	Low	Unclear	Unclear	Moderate
Ye QM 2022	Low	Unclear	High	Unclear	Low	Unclear	Unclear	Moderate

Young HN 2012	Unclear	Low	High	Unclear	Low	Unclear	Unclear	Moderate
Yu JK 2023	Low	Unclear	High	Unclear	Low	Unclear	Unclear	Moderate
Zhang W 2022	Unclear	Unclear	High	Unclear	Low	Unclear	Unclear	High
Zhang XS 2019	Unclear	Unclear	High	Unclear	Low	Low	Unclear	High
Zhang YL 2023	Unclear	Unclear	High	Unclear	Low	Unclear	Unclear	High
Zhao JY 2023	Unclear	Unclear	High	Unclear	Low	Unclear	Unclear	Moderate

4.2 Risk of bias graph

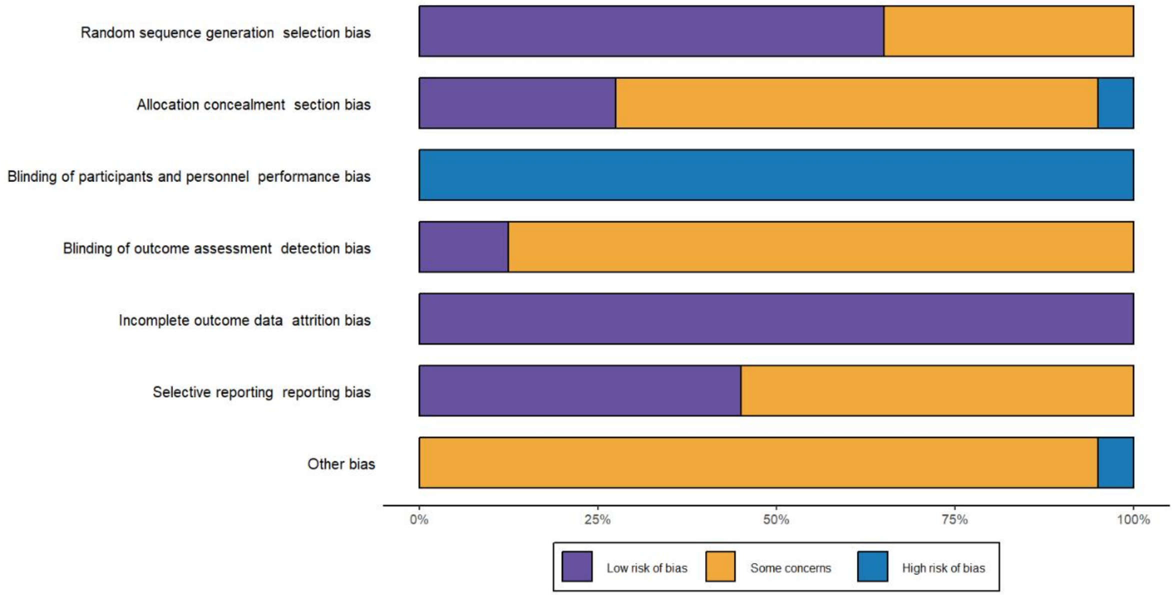

Supplement: Multimedia Appendix 4 [file jmir_v27i1e64073_app4.pdf]
