# Supplementary material for: Evaluating the Impact of the COVID-19 Pandemic on Telepharmaceutical Service Effectiveness: Systematic Review and Meta-Analysis
Source: J Med Internet Res. 2025 Jul 2;27:e64073. doi: 10.2196/64073 (PMC12268221; doi:10.2196/64073)
Supplement: Multimedia Appendix 5 [file jmir_v27i1e64073_app5.pdf]

Multimedia Appendix 5: GRADE for evidence

Outcomes	Subgroup	Study characteristic	Absolute effect (RD/MD)	Risk of bias	Indirectness	Inconsistency	Imprecision	Other consideration	Rating
Medication adherence (D)	Overall	3387 patients from 14 studies	0.15 (0.09 to 0.20)	Serious[a]	Not serious	Not serious	Not serious	Not serious	Moderate
	Before the outbreak	2425 patients from 9 studies	0.11 (0.05 to 0.16)	Not serious	Not serious	Not serious	Not serious	Not serious	High
	After the outbreak	962 patients from 5 studies	0.23 (0.16 to 0.29)	Serious[a]	Not serious	Not serious	Not serious	Not serious	Moderate
Medication adherence (C)	Overall	6917 patients from 7 studies	14.03 (7.37 to 20.69)	Serious[a]	Not serious	Not serious	Not serious	Not serious	Moderate
	Before the outbreak	6004 patients from 4 studies	12.26 (0.38 to 24.14)	Not serious	Not serious	Serious[c]	Not serious	Not serious	Moderate
	After the outbreak	913 patients from 3 studies	16.44 (15.37 to 17.52)	Serious[a]	Not serious	Not serious	Not serious	Not serious	Moderate
Medication satisfaction (D)	Overall	2433 patients from 6 studies	0.16 (0.05 to 0.26)	Serious[a]	Not serious	Serious[c]	Not serious	Not serious	Low
	Before the outbreak	2221 patients from 4 studies	0.17 (0.03 to 0.30)	Serious[a]	Not serious	Serious[c]	Not serious	Not serious	Low
	After the outbreak	212 patients from 2 studies	0.15 (-0.10 to 0.39)	Very serious[b]	Not serious	Serious[c]	Serious[d]	Not serious	Very low
Medication satisfaction (C)	Overall	406 patients from 2 studies	3.73 (-1.96 to 9.43)	Serious[a]	Not serious	Not serious	Serious[d]	Not serious	Low

**Outcomes**	**Subgroup**	**Study characteristic**	**Absolute effect (RD/MD)**	**Risk of bias**	**Indirectness**	**Inconsistency**	**Imprecision**	**Other consideration**	**Rating**
	Before the outbreak	406 patients from 2 studies	3.73 (-1.96 to 9.43)	Serious[a]	Not serious	Not serious	Serious[d]	Not serious	Low
Adverse events (D)	Overall	2756 patients from 11 studies	-0.10 (-0.18 to -0.02)	Serious[a]	Not serious	Serious[c]	Not serious	Not serious	Low
	Before the outbreak	872 patients from 4 studies	-0.09 (-0.34 to 0.15)	Not serious	Not serious	Serious[c]	Serious[d]	Not serious	Low
	After the outbreak	1884 patients from 7 studies	-0.08 (-0.12 to -0.05)	Serious[a]	Not serious	Not serious	Not serious	Not serious	Moderate
Diabetes									
HbA_1c (C)	Overall	546 patients from 5 studies	-0.48 (-0.93 to -0.03)	Not serious	Not serious	Not serious	Serious[e]	Not serious	Moderate
	Before the outbreak	197 patients from 2 studies	-0.37 (-1.02 to 0.28)	Not serious	Not serious	Not serious	Serious[d]	Not serious	Moderate
	After the outbreak	349 patients from 2 studies	-0.66 (-1.62 to 0.31)	Not serious	Not serious	Not serious	Serious[d]	Not serious	Moderate
FBG (C)	Overall	359 patients from 2 studies	-1.12 (-2.69 to 0.45)	Not serious	Not serious	Serious[c]	Serious[d]	Not serious	Low
	Before the outbreak	119 patients from 1 study	-0.33 (-0.69 to 0.03)	Not serious	Not serious	Not serious	Serious[d]	Not serious	Moderate
	After the outbreak	240 patients from 1 study	-1.93 (-2.41 to -1.45)	Not serious	Not serious	Not serious	Serious[e]	Not serious	Moderate
2h PG (C)	Overall	240 patients from 1 study	-2.77 (-3.47 to -2.07)	Not serious	Not serious	Not serious	Serious[e]	Not serious	Moderate

Outcomes	Subgroup	Study characteristic	Absolute effect (RD/MD)	Risk of bias	Indirectness	Inconsistency	Imprecision	Other consideration	Rating
	After the outbreak	240 patients from 1 study	-2.77 (-3.47 to -2.07)	Not serious	Not serious	Not serious	Serious[e]	Not serious	Moderate
Hypertension									
SBP (C)	Overall	2182 patients from 5 studies	-5.03 (-9.10 to -0.95)	Not serious	Not serious	Serious[c]	Not serious	Not serious	Moderate
	Before the outbreak	1869 patients from 3 studies	-3.82 (-10.63 to 2.99)	Not serious	Not serious	Serious[c]	Serious[d]	Not serious	Low
	After the outbreak	313 patients from 2 studies	-7.49 (-7.79 to -7.18)	Serious[a]	Not serious	Not serious	Serious[e]	Not serious	Low
DBP (C)	Overall	1167 patients from 4 studies	-4.13 (-4.40 to -3.85)	Not serious	Not serious	Not serious	Not serious	Not serious	High
	Before the outbreak	854 patients from 2 studies	-3.98 (-5.48 to -2.47)	Not serious	Not serious	Not serious	Not serious	Not serious	High
	After the outbreak	313 patients from 2 studies	-5.57 (-9.02 to -2.12)	Serious[a]	Not serious	Not serious	Serious[e]	Not serious	Low
Anticoagulation									
INR (2 to 3) (D)	Overall	560 patients from 3 studies	0.07 (-0.06 to 0.21)	Serious[a]	Not serious	Serious[c]	Serious[d]	Not serious	Low
	Before the outbreak	560 patients from 3 studies	0.07 (-0.06 to 0.21)	Serious[a]	Not serious	Serious[c]	Serious[d]	Not serious	Low
TTR (C)	Overall	857 patients from 4 studies	12.97 (5.02 to 20.92)	Serious[a]	Not serious	Not serious	Not serious	Not serious	Moderate
	Before the outbreak	857 patients	12.97	Serious[a]	Not serious	Not serious	Not serious	Not serious	Moderate

**Outcomes**	**Subgroup**	**Study characteristic**	**Absolute effect (RD/MD)**	**Risk of bias**	**Indirectness**	**Inconsistency**	**Imprecision**	**Other consideration**	**Rating**
		from 4 studies	(5.02 to 20.92)						
Stroke									
Recurrence of stroke (D)	Overall	60 patients from 1 study	-0.20 (-0.38 to -0.02)	Not serious	Not serious	Not serious	Very serious[f]	Not serious	Low
	After the outbreak	60 patients from 1 study	-0.20 (-0.38 to -0.02)	Not serious	Not serious	Not serious	Very serious[f]	Not serious	Low
BI (C)	Overall	60 patients from 1 study	14.75 (8.78 to 20.72)	Not serious	Not serious	Not serious	Very serious[f]	Not serious	Low
	After the outbreak	60 patients from 1 study	14.75 (8.78 to 20.72)	Not serious	Not serious	Not serious	Very serious[f]	Not serious	Low
Cancer									
Cancer pain (C)	Overall	60 patients from 1 study	-2.39 (-2.56 to -2.22)	Serious[a]	Not serious	Not serious	Very serious[f]	Not serious	Very low
	Before the outbreak	60 patients from 1 study	-2.39 (-2.56 to -2.22)	Serious[a]	Not serious	Not serious	Very serious[f]	Not serious	Very low
Respiratory diseases									
FEV_1 (C)	Overall	164 patients from 1 study	8.51 (6.77 to 10.25)	Not serious	Not serious	Not serious	Very serious[f]	Not serious	Low
	Before the outbreak	164 patients from 1 study	8.51 (6.77 to 10.25)	Not serious	Not serious	Not serious	Very serious[f]	Not serious	Low
PEF (C)	Overall	678 patients from 2 studies	88.66 (60.03 to 117.29)	Not serious	Not serious	Not serious	Serious[e]	Not serious	Moderate

Outcomes	Subgroup	Study characteristic	Absolute effect (RD/MD)	Risk of bias	Indirectness	Inconsistency	Imprecision	Other consideration	Rating
	Before the outbreak	678 patients from 2 studies	88.66 (60.03 to 117.29)	Not serious	Not serious	Not serious	Serious[e]	Not serious	Moderate
Respiratory function (C)	Overall	514 patients from 1 study	-0.69 (-0.72 to -0.66)	Not serious	Not serious	Not serious	Serious[e]	Not serious	Moderate
	Before the outbreak	514 patients from 1 study	-0.69 (-0.72 to -0.66)	Not serious	Not serious	Not serious	Serious[e]	Not serious	Moderate

Abbreviations: D (dichotomous outcome); C (continuous outcome); RR (risk ratio); RD (risk difference); MD (mean difference); HbA_1c (Hemoglobin A_1c); FBG (fasting blood glucose); 2h PG (2-hour post prandial blood glucose); SBP (systolic blood pressure); DBP (diastolic blood pressure); INR (international normalized ratio); TTR (time in therapeutic range); BI (Barthel index); FEV_1 (forced expiratory volume in one second); PEF (peak expiratory flow).

a. Risk of bias: Serious. The risk or bias of almost every study contributing to the outcomes was serious.

b. Risk of bias: Very serious. The risk or bias of almost every study contributing to the outcomes was very serious.

c. Inconsistency: Serious. The effect sizes between studies were not similar and considering heterogeneity with bigger I^2 (>50%).

d. Imprecision: Serious. Downgraded due to wide confidence intervals crossing the null.

e. Imprecision: Serious. Downgraded due to sample size which is smaller than the optimal information size (OIS).

f. Imprecision: Very serious. Downgraded due to sample size which is smaller than 30-50% of the OIS.
