# Supplementary material for: Evaluating the Impact of the COVID-19 Pandemic on Telepharmaceutical Service Effectiveness: Systematic Review and Meta-Analysis
Source: J Med Internet Res. 2025 Jul 2;27:e64073. doi: 10.2196/64073 (PMC12268221; doi:10.2196/64073)
Supplement: Multimedia Appendix 6 [file jmir_v27i1e64073_app6.pdf]

Supplementary material 6: The independent sample *z* test strategies and methods

We applied independent sample *z* test to compare the difference between the subgroups (before and after the outbreak of the COVID-19 pandemic).[1, 2] If μ_1 and μ_2 stand for the absolute effect size (RD/MD) respectively:

The difference in the effect size between subgroups: $diff = \mu_1 - \mu_2$

Standard error: $se_{diff} = \sqrt{se_1^2 + se_2^2}$

95% confidence interval: $diff \pm 1.96 \times se_{diff}$

z value: $z = \frac{diff}{se_{diff}}$

z follows a normal distribution, $P = 2[1 - \emptyset(|z|)]$

The codes in R studio were listed as follow: (take continuous data for example)

Loading packages<br><br>library(metafor)
Reading data named 'condata' and calculating MD and 95% CI for each study<br><br>data <-read.csv("C:/1.csv")<br><br>condata<-escalc(m1i=m1, sd1=sd1, n1i=n1, m2i=m2, sd2i=sd2, n2i=n2,measure="MD", data=data)
The independent sample z test
 # Performing meta-analyses with random-effect model and pooling effect sizes for each subgroup<br><br>meta<-rma(yi, vi, data=condata)<br><br>meta1<-rma(yi, vi, data=condata, subset=Subgroup=="After the outbreak")<br><br>meta2<-rma(yi, vi, data=condata, subset=Subgroup=="Before the outbreak")<br><br>summary(meta)<br><br>summary(meta1)<br><br>summary(meta2)
 # Calculating the difference in the effect size between subgroups, standard error, and 95% CI<br><br>diffes<-coef(meta1)-coef(meta2)<br><br>diffesse<-sqrt((meta1$se)^2+(meta2$se)^2)<br><br>diffesll<-diffes-1.96*diffesse<br><br>diffesul<-diffes+1.96*diffesse
 # Calculating the z value and P value

```
zvalue<-diffes/diffesse

pvalue<-round(2*pnorm(-abs(zvalue)),4)
```

```
# Displaying the relevant values

diffes

diffesll

diffesul

zvalue

pvalue
```

Reference

1. Higgins JP, Deeks JJ. Selecting Studies and Collecting Data, eds. Cochrane handbook for systematic reviews of interventions. Wiley-Blackwell, 2008: 151-185. https://onlinelibrary.wiley.com/doi/abs/10.1002/9780470712184.ch7 [accessed 2024-07-09]
2. Zhang TS, Zhang SX. How to implement the comparison of different subcombinations and effect sizes in Meta-analysis. Chin J Evid Based Med 2017; 17(12): 1465-1470.[In Chinese]. https://kns.cnki.net/kcms2/article/abstract?v=VKFFl0Cm57b3tb2aYmxV1hR_Z2UAJ-h378z36o32XXFkTr9foSQlc_HtpDaQnzI8yQ0zQrOfY_yIBdqXlJn65q4EeVVxPF5C-fI8V8o6Auut7Sy4t2Hmde6OOmdCkUfYbZiwXpejoTJoCeUrbeBxBg==&uniplatform=NZKPT&language=CHS [accessed 2024-07-09]
