# Supplementary material for: Evaluating the Impact of the COVID-19 Pandemic on Telepharmaceutical Service Effectiveness: Systematic Review and Meta-Analysis
Source: J Med Internet Res. 2025 Jul 2;27:e64073. doi: 10.2196/64073 (PMC12268221; doi:10.2196/64073)
Supplement: Multimedia Appendix 7 [file jmir_v27i1e64073_app7.pdf]

Multimedia Appendix 7: Summary of findings

Outcomes	Subgroup	Number of studies	Number of participants	Relative effect[a] (RR) (95% CI)	Absolute effect (RD/MD) (95% CI)	Certainty of evidence
Medication adherence (D)	Overall	14	3387	1.19 (1.11 to 1.27)	0.15 (0.09 to 0.20)	Moderate
	Before the outbreak	9	2425	1.12 (1.05 to 1.20)	0.11 (0.05 to 0.16)	High
	After the outbreak	5	962	1.31 (1.19 to 1.43)	0.23 (0.16 to 0.29)	Moderate
Medication adherence[b] (C)	Overall	7	6917	N/A	14.03 (7.37 to 20.69)	Moderate
	Before the outbreak	4	6004	N/A	12.26 (0.38 to 24.14)	Moderate
	After the outbreak	3	913	N/A	16.44 (15.37 to 17.52)	Moderate
Medication satisfaction (D)	Overall	6	2433	1.23 (1.07 to 1.42)	0.16 (0.05 to 0.26)	Low
	Before the outbreak	4	2221	1.26 (1.05 to 1.51)	0.17 (0.03 to 0.30)	Low
	After the outbreak	2	212	1.19 (0.87 to 1.64)	0.15 (-0.10 to 0.39)	Very low
Medication satisfaction (C)	Overall	2	406	N/A	3.73 (-1.96 to 9.43)	Low
	Before the outbreak	2	406	N/A	3.73 (-1.96 to 9.43)	Low
Adverse events (D)	Overall	11	2756	0.59 (0.36 to 0.97)	-0.10 (-0.18 to -0.02)	Low
	Before the outbreak	4	872	0.60 (0.13 to 2.86)	-0.09 (-0.34 to 0.15)	Low
	After the outbreak	7	1884	0.64 (0.53 to 0.79)	-0.08 (-0.12 to -0.05)	Moderate
Diabetes						
HbA_1c (C)	Overall	4	546	N/A	-0.48 (-0.93 to -0.03)	Moderate
	Before the outbreak	2	197	N/A	-0.37 (-1.02 to 0.28)	Moderate
	After the outbreak	2	349	N/A	-0.66 (-1.62 to 0.31)	Moderate
FBG[c] (C)	Overall	2	359	N/A	-1.12 (-2.69 to 0.45)	Low
	Before the outbreak	1	119	N/A	-0.33 (-0.69 to 0.03)	Moderate
	After the outbreak	1	240	N/A	-1.93 (-2.41 to -1.45)	Moderate
2h PG (C)	Overall	1	240	N/A	-2.77 (-3.47 to -2.07)	Moderate

Outcomes	Subgroup	Number of studies	Number of participants	Relative effect[a] (RR) (95% CI)	Absolute effect (RD/MD) (95% CI)	Certainty of evidence
	After the outbreak	1	240	N/A	-2.77 (-3.47 to -2.07)	Moderate
Hypertension						
SBP (C)	Overall	5	2182	N/A	-5.03 (-9.10 to -0.95)	Moderate
	Before the outbreak	3	1869	N/A	-3.82 (-10.63 to 2.99)	Low
	After the outbreak	2	313	N/A	-7.49 (-7.79 to -7.18)	Low
DBP (C)	Overall	4	1167	N/A	-4.13 (-4.40 to -3.85)	High
	Before the outbreak	2	854	N/A	-3.98 (-5.48 to -2.47)	High
	After the outbreak	2	313	N/A	-5.57 (-9.02 to -2.12)	Low
Anticoagulation[d]						
INR (2 to 3) (D)	Overall	3	560	1.21 (0.84 to 1.75)	0.07 (-0.06 to 0.21)	Low
	Before the outbreak	3	560	1.21 (0.84 to 1.75)	0.07 (-0.06 to 0.21)	Low
TTR (C)	Overall	4	857	N/A	12.97 (5.02 to 20.92)	Moderate
	Before the outbreak	4	857	N/A	12.97 (5.02 to 20.92)	Moderate
Stroke						
Recurrence of stroke (D)	Overall	1	60	0.25 (0.06 to 1.08)	-0.20 (-0.38 to -0.02)	Low
	After the outbreak	1	60	0.25 (0.06 to 1.08)	-0.20 (-0.38 to -0.02)	Low
BI (C)	Overall	1	60	N/A	14.75 (8.78 to 20.72)	Low
	After the outbreak	1	60	N/A	14.75 (8.78 to 20.72)	Low
Cancer						
Cancer pain[e] (C)	Overall	1	60	N/A	-2.39 (-2.56 to -2.22)	Very low
	Before the outbreak	1	60	N/A	-2.39 (-2.56 to -2.22)	Very low
Respiratory diseases						
FEV_1 (C)	Overall	1	164	N/A	8.51 (6.77 to 10.25)	Low
	Before the outbreak	1	164	N/A	8.51 (6.77 to 10.25)	Low

Outcomes	Subgroup	Number of studies	Number of participants	Relative effect[a] (RR) (95% CI)	Absolute effect (RD/MD) (95% CI)	Certainty of evidence
PEF[f] (C)	Overall	2	678	N/A	88.66 (60.03 to 117.29)	Moderate
	Before the outbreak	2	678	N/A	88.66 (60.03 to 117.29)	Moderate
Respiratory function[g] (C)	Overall	1	514	N/A	-0.69 (-0.72 to -0.66)	Moderate
	Before the outbreak	1	514	N/A	-0.69 (-0.72 to -0.66)	Moderate

Abbreviations: D (dichotomous outcome); C (continuous outcome); RR (risk ratio); RD (risk difference); MD (mean difference); HbA_1c (Hemoglobin A_1c); FBG (fasting blood glucose); 2h PG (2-hour post prandial blood glucose); SBP (systolic blood pressure); DBP (diastolic blood pressure); INR (international normalized ratio); TTR (time in therapeutic range); BI (Barthel index); FEV_1 (forced expiratory volume in one second); PEF (peak expiratory flow).

[a] N/A stood for relative effect not applicable for continuous outcomes.

[b] The measurement scale for medication adherence and satisfaction were converted to a 100-point scale on a uniform basis.

[c] A study (Zhou C 2020) reported FPG (fasting plasma glucose) other than FBG. But it may be a potential error in FBG as FPG through our confirming article.

[d] The patients took warfarin for anticoagulation

[e] The study measured pain through numerical rating scale (NRS).

[f] A study (Xu JY 2023) reported PEF both in morning and evening, and we selected morning for statistic.

[g] The study (Xu JY 2023) measured respiratory function through modified British medical research council (mMRC) scale.
