# Supplementary material for: Evaluating the Impact of the COVID-19 Pandemic on Telepharmaceutical Service Effectiveness: Systematic Review and Meta-Analysis
Source: J Med Internet Res. 2025 Jul 2;27:e64073. doi: 10.2196/64073 (PMC12268221; doi:10.2196/64073)
Supplement: Multimedia Appendix 8 [file jmir_v27i1e64073_app8.pdf]

Multimedia Appendix 8: Characteristics of included studies

Author & publication year	Study setting				Participants characteristic				Description of TPS
	Conduction year [a]	Country	Regions	Income levels	Description of participants	Sample size (N) [c]	Participants age (mean)	Female (N, %)	
**Before the outbreak of the COVID-19 pandemic**									
Bynum A 2001	1998	United States	North America	High income	Adolescents in grades 7–12 with asthma in rural Arkansas	49	12–14: 20<br>15–17: 23<br>18-19: 3<br>Nonrespondents: 3	34 (69.4%)	Telepharmacy counseling provided accessible education regarding metered-dose inhaler technique by interactive compressed video.
Staresinic AG 2006	1999	United States	North America	High income	Adults receiving long-term warfarin therapy	192	69.3	5 (2.6%)	Fastidious telephone-based telepharmaceutical contact following each clinic visit.
Elliott RA 2008	2004	United Kingdom	Europe & Central Asia	High income	Patients receiving a new medicine for a chronic condition	205	67.0	110 (53.7%)	Pharmacists gave adviceand enquired about any medicine-related problems, adherence to the medicine and whether they required any further information via telephone.
Green BB 2008	2005	United States	North America	High income	Patients with uncontrolled essential hypertension and taking antihypertensive	519 [d]	59.0	287 (55.3%)	Home blood pressure monitoring and Web training plus pharmacist care management delivered through Web communications.

Author & publication year	Study setting				Participants characteristic				Description of TPS
	Conduction year [a]	Country	Regions	Income levels	Description of participants	Sample size (N) [c]	Participants age (mean)	Female (N, %)	
					medications				
Ralston JD 2014 [e]	2005	United States	North America	High income	Patients with a hypertension diagnosis and taking antihypertensive medications	383	59.8	191 (49.9%)	Pharmacists assisted care with home blood pressure monitor delivered over the patient website.
Magid DJ 2011	2006	United States	North America	High income	Patients with uncontrolled blood pressure	283	65.9	183 (35.3%)	Patient education, home blood pressure monitoring, blood pressure measurement reporting to an interactive voice response phone system, and clinical pharmacist follow-up.
Magid DJ 2013	2008	United States	North America	High income	Patients with blood pressure above recommended levels[e]	348	59.6	138 (39.7%)	Pharmacist-led, Heart360-enabled, home blood pressure monitoring intervention. Pharmacists reviewed patients' current blood pressure medication regimen, provided counseling on lifestyle changes, and adjusted or changed antihypertensive medications as needed.
Alsabbagh MW 2012	2009	Canada	North America	High income	Patients being discharged from	94	63.0	20 (21.3%)	Pharmacists telephoned patients to identify the barriers to optimal

Author & publication year	Study setting				Participants characteristic				Description of TPS
	Conduction year [a]	Country	Regions	Income levels	Description of participants	Sample size (N) [c]	Participants age (mean)	Female (N, %)	
					hospitals after an acute coronary syndrome or revascularization				utilization of drugs or adherence. Then pharmacists proposed the date of the next call within 1 to 2 weeks, according to the need to support medication adherence including education on side effects or intolerance, cost concerns, and drug interactions.
Margolis KL 2013	2009	United States	North America	High income	Patients with uncontrolled hypertension	450	61.1	201 (44.7%)	Combining home blood pressure telemonitoring with pharmacist case management.
Young HN 2012	2009	United States	North America	High income	Patients with asthma who received their medications	98	44.6	75 (76.5%)	Telephone consultations from trained pharmacists regarding asthma self-management and medication use
Lyons I 2016	2012	United Kingdom	Europe & Central Asia	High income	Patients prescribed at least one oral medication for type 2 diabetes and/or lipid regulation	677	69.9	281 (41.5%)	Tailored telephone consultations with a pharmacist and written summary of the discussion and a medicines reminder chart.
Choudhry KN 2018	2015	United States	North America	High income	Patients with suboptimal hyperlipidemia,	4078[g]	59.8	1841 (45.1%)	A multicomponent intervention using telephone-delivered behavioral interviewing by

Author & publication year	Study setting				Participants characteristic				Description of TPS
	Conduction year [a]	Country	Regions	Income levels	Description of participants	Sample size (N) [c]	Participants age (mean)	Female (N, %)	
					hypertension, or diabetes disease control, and who were nonadherent to prescribed medications for these conditions				trained clinical pharmacists, text messaging, pillboxes, and mailed progress reports.
Shi NN 2021	2016	China	East Asia & Pacific	Upper middle income	Discharged elderly patients with hypertension	93	70.0	48 (51.6%)	Clinical pharmacists provided remote medication guidance and pharmaceutical care.
Chen Y 2017	2016	China	East Asia & Pacific	Upper middle income	Chronic patients who need long-term medication	400	61.5	171 (42.8%)	Pharmacists conducted follow-up services and medication guidance by video and reminded patients to take medication through a computer system
Lu Y 2017	2016	China	East Asia & Pacific	Upper middle income	Chronic patients who need long-term medication	400	62.0	168 (42.0%)	Telepharmaceutical services including video calls, medication guidance and medication schedules.
Lauffenburger JC 2019	2016	United States	North America	High income	Adult patients with poorly-controlled diabetes	1400	54.8	521 (37.2%)	Telephone-based telepharmaceutical services that integrated brief negotiated interviewing and shared

Author & publication year	Study setting				Participants characteristic				Description of TPS
	Conduction year [a]	Country	Regions	Income levels	Description of participants	Sample size (N) [c]	Participants age (mean)	Female (N, %)	
									decision-making to identify patient goals and options for enhancing diabetes management.
Sudas Na Ayutthaya N 2018	2016	Thailand	East Asia & Pacific	Upper middle income	Patients aged ≥ 20 years who were prescribed warfarin when discharged	50	57.7	30 (60.0%)	During each 10–25-minute telephone call, pharmacists performed medicine use review by asking patients about problems/obstacles with managing warfarin including adverse events and complications, assessing medication adherence, and giving reminders for the next scheduled visits. Pharmacists promptly addressed any problems identifed during the telephone call with patients.
Margolis KL 2022	2017	United States	North America	High income	Adult patients with moderately severe hypertension (BP ≥ 150/95 mmHg)	3071	60.2	1639 (53.4%)	Telehealth care using best practices and adding home blood pressure telemonitoring with home-based care coordinated by a clinical pharmacist or nurse practitioner (limited MTM pharmacist capacity).

Author & publication year	Study setting				Participants characteristic				Description of TPS
	Conduction year [a]	Country	Regions	Income levels	Description of participants	Sample size (N) [c]	Participants age (mean)	Female (N, %)	
Xu JY 2023	2018	China	East Asia & Pacific	Upper middle income	Adult patients with chronic obstructive pulmonary disease admitted to hospital	570	63.2	289 (50.7%)	Telepharmaceutical services including medication plan,timely medication reminder, medication record and other guidance provided by pharmacists through mobile.
Wan JW 2022	2018	China	East Asia & Pacific	Upper middle income	Adult patients treated with warfarin admitted to the hospital	210	67.4	111 (52.9%)	Clinical pharmacists conducted warfarin anticoagulation management for patients through Wechat.
Zhang XS 2019	2018	China	East Asia & Pacific	Upper middle income	Diabetic patients receiving medicine at outpatient clinic	240	41-61	108 (45.0%)	Pharmacists tracked patients' blood glucose, timely adjust and optimize drug use plans, remind patients to take drugs, provide relevant drug information with the help of telematics management systems, and regularly communicate with patients by phone.
Lu ZW 2021	2019	China	East Asia & Pacific	Upper middle income	Patients from the inpatient department of the hospital. The main reasons for	119	55.0	54 (45.4%)	Internet-based medication management service including in-hospital pharmacy consultation, medication

Author & publication year	Study setting				Participants characteristic				Description of TPS
	Conduction year [a]	Country	Regions	Income levels	Description of participants	Sample size (N) [c]	Participants age (mean)	Female (N, %)	
					admission were poor recent blood glucose control, requests for diabetes complications tests, and frequent hypoglycemic events recently				evaluation, and treatment and out-of-hospital medication consultation, guidance, and follow-up.
Zhao JY 2023	2019	China	East Asia & Pacific	Upper middle income	Hospitalized patients with bronchial asthma	164	53.8	66 (40.2%)	Medication consultation, medication schedule planning, medication education and medication reminder through internet.
Li JY 2022	2019	China	East Asia & Pacific	Upper middle income	Hospitalized patients taking warfarin	405	50.8	189 (46.7%)	Medication consultation and anticoagulation management through application by mobile after discharge.
Jin X 2021	2019	China	East Asia & Pacific	Upper middle income	Patients with chronic obstructive pulmonary disease	80	57.9	35 (43.8%)	Internet-based medication management service through application by mobile.
Feng H 2021	2019	China	East Asia & Pacific	Upper middle income	Patients with cancer	60	Not reported	25 (41.7%)	Medication consultation, medication education and psychological counseling by

Author & publication year	Study setting				Participants characteristic				Description of TPS
	Conduction year [a]	Country	Regions	Income levels	Description of participants	Sample size (N) [c]	Participants age (mean)	Female (N, %)	
									Wechat group.
Peasah SK 2020	Not reported [b]	United States	North America	High income	Patients aged 18 to 65 years old taking at least 1 oral antidiabetic medication, and had an HbA_1c within the last 12 months of ≥ 7%.	78	61.7	37 (47.4%)	Telephone follow-up intervention regarding diabetic medication adherence by student pharmacists.
**After the outbreak of the COVID-19 pandemic**									
Yu JK 2023	2020	China	East Asia & Pacific	Upper middle income	Adult patients with asthma and chronic obstructive pulmonary disease diagnosed in the outpatient clinic of pulmonary and critical care medicine	286	48.9	132 (46.1%)	Internet medication consultation and remote follow-up management by pharmacists.
Ye QM 2022	2020	China	East Asia & Pacific	Upper middle income	Hospitalized patients with ischemic stroke	60	69.3	48 (80%)	Medication consultation and medication reminder on the internet after discharge.
Wang ZM(1)	2020	China	East Asia &	Upper middle	Elderly patients with coronary heart	600	72.7	273 (45.5%)	Medication counseling, medication recombination,

Author & publication year	Study setting				Participants characteristic				Description of TPS
	Conduction year [a]	Country	Regions	Income levels	Description of participants	Sample size (N) [c]	Participants age (mean)	Female (N, %)	
2023			Pacific	income	disease who take medication at home				reminders, adverse reaction monitoring and medication education on the internet by clinical pharmacists.
Wang ZM(2) 2023	2020	China	East Asia & Pacific	Upper middle income	Elderly patients with type 2 diabetes mellitus	600	64.3	224 (37.3%)	Medication guidance and providing medication knowledge by telephone and video call.
Liu Y 2022	2020	China	East Asia & Pacific	Upper middle income	Patients with breast cancer who receive endocrine therapy	92	50.4	92 (100.0%)	Medication consultation and medication education on the internet.
Jiang H 2022	2020	China	East Asia & Pacific	Upper middle income	Elderly patients with hypertension	80	70.7	25 (31.3%)	Remote follow-up by telephone and Wechat and medication consultation.
Ibrahim OM 2022	2021	United Arab Emirates	Middle East & North Africa	High income	Adult patients with uncontrolled hypertension	239	60.9	107 (44.8%)	Telepharmaceutical services delivered by community pharmacies including reviewing medications, detecting any drug-related problems, and making necessary adjustments during the tele-meeting between pharmacists and patients.
Zhang YL 2023	2021	China	East Asia &	Upper middle	Homebound patients with psychiatric	132	42.6	63 (47.7%)	Pharmacists use WeChat to explain medication precautions,

Author & publication year	Study setting				Participants characteristic				Description of TPS
	Conduction year [a]	Country	Regions	Income levels	Description of participants	Sample size (N) [c]	Participants age (mean)	Female (N, %)	
			Pacific	income	diseases				knowledge related to adverse reactions and treatment of adverse reactions and conduct remote follow-up visits.
Zhang W 2022	2021	China	East Asia & Pacific	Upper middle income	Discharged patients with stroke	90	59.2	41 (45.6%)	Providing medication-related knowledge, adjusting medication, and remote follow-up by WeChat.
Liu H 2022	2021	China	East Asia & Pacific	Upper middle income	Patients with ovarian cancer who took poly ADP ribose polymerase inhibitor orally	54	≤ 35: 2<br>36-50: 6<br>51-60: 28<br>≥ 61: 18	54 (100.0%)	Providing medication-related knowledge and remote medication education by WeChat group.
Liao QQ 2023	2021	China	East Asia & Pacific	Upper middle income	Patients with type 2 diabetes mellitus	120	71.1	58 (48.3%)	Providing medication-related knowledge by WeChat, communication with patients by WeChat and telephone, remote follow-up by telephone, and medication reminder by short message service.
Chen XQ 2022	2021	China	East Asia & Pacific	Upper middle income	Patients aged more than 40 years old with diabetes	240	60.0	80 (33.3%)	Providing medication-related knowledge, medication management, and contacting with patients whose blood glucose is

Author & publication year	Study setting				Participants characteristic				Description of TPS
	Conduction year [a]	Country	Regions	Income levels	Description of participants	Sample size (N) [c]	Participants age (mean)	Female (N, %)	
									not meeting expectations to develop medication plans.
Khan YH 2022	2021	Saudi Arabia	Middle East & North Africa	High income	Patients aged more than 30 years old with type 2 diabetes mellitus	109	58.3	66 (60.6%)	Pharmacists provided telepharmaceutical services (calls, messages, or emails) to patients in order to cater their medication-related problems, inquire about medication adherence and follow-up. Telepharmaceutical services also included pictorial messages to patients that focused primarily on the effective use of diabetes medicine, maintaining a predefined glycemic control and eating habits.

a. Whey identified the start year of RCTs on the clinical trials registered platform (https://classic.clinicaltrials.gov/ct2/) when no information in the study about the start year.

b. We classified the studies to before the outbreak of the COVID-19 pandemic according to the publishing year of studies because of no available information.

c. We recorded the number of participants in the baseline characteristic reported by author.

d. This is a three-arms trial and we selected two arms (TPS versus usual care) of it.

e. This is a mediation analysis of a published randomized trial based on the Chronic Care Model (Green BB 2008), but they analyzed different outcomes. We included medication adherence in Ralston JD 2014 and we included systolic blood pressure and diastolic blood pressure in Green BB 2008.

f. Recommend levels: systolic BP [SBP] ≥ 140 mmHg or diastolic BP [DBP] ≥ 90 mmHg or, for those with diabetes mellitus or chronic kidney disease, SBP ⩾130 mmHg

Author & publication year	Study setting				Participants characteristic				Description of TPS
	Conduction year [a]	Country	Regions	Income levels	Description of participants	Sample size (N) [c]	Participants age (mean)	Female (N, %)	
or DBP ⩾80 mmHg<br>g. The number of participants: hyperlipidemia: 2970; hypertension: 1015; diabetes: 488									
