# Supplementary material for: Evaluating the Impact of the COVID-19 Pandemic on Telepharmaceutical Service Effectiveness: Systematic Review and Meta-Analysis
Source: J Med Internet Res. 2025 Jul 2;27:e64073. doi: 10.2196/64073 (PMC12268221; doi:10.2196/64073)
Supplement: Multimedia Appendix 9 [file jmir_v27i1e64073_app9.pdf]

Multimedia Appendix 9: Classification and characteristic of TPS

Author & publication year	Medication selection and recommendation	Pharmaceutical counseling	Medication monitoring (effectiveness and side effect)	Medication adherence assessments	Medication guidance	Health management (lifestyle)
**Intervention 1: TPS by Pharmacists**						
Alsabbagh MW 2012			√	√		
Bynum A 2001		√			√	
Chen XQ 2022	√	√	√	√	√	√
Chen Y 2017		√	√	√	√	
Choudhry KN 2018				√		
Elliott RA 2008			√	√		
Feng H 2021		√		√	√	√
Ibrahim OM 2022	√		√	√		
Jiang H 2022		√	√		√	√
Jin X 2021	√	√				
Khan YH 2022		√		√	√	√
Lauffenburger JC 2019				√		√
Li JY 2022		√	√	√		
Liao QQ 2023			√		√	
Liu H 2022		√	√	√	√	√
Liu Y 2022		√	√		√	
Lu Y 2017		√	√	√	√	
Lu ZW 2021	√	√	√	√	√	√
Lyons I 2016			√	√		√

Author & publication year	Medication selection and recommendation	Pharmaceutical counseling	Medication monitoring (effectiveness and side effect)	Medication adherence assessments	Medication guidance	Health management (lifestyle)
Peasah SK 2020			√	√		
Shi NN 2021		√	√	√	√	√
Staresinic AG 2006						
Sudas Na Ayutthaya N 2018		√	√	√		
Wan JW 2022	√	√	√	√	√	
Wang ZM(1) 2023		√	√	√	√	
Wang ZM(2) 2023			√	√	√	
Xu JY 2023		√	√		√	
Ye QM 2022		√	√	√		√
Young HN 2012		√	√			
Yu JK 2023		√	√	√	√	√
Zhang W 2022	√		√	√	√	√
Zhang XS 2019		√	√	√		√
Zhang YL 2023			√	√	√	√
Zhao JY 2023		√	√	√	√	√
**Intervention 2: TPS by pharmacists and telemonitoring device**						
Green BB 2008	√	√				√
Magid DJ 2011	√	√	√			√
Magid DJ 2013	√	√	√	√		√
Margolis KL 2013	√		√	√	√	√
Margolis KL 2022		√	√			

Author & publication year	Medication selection and recommendation	Pharmaceutical counseling	Medication monitoring (effectiveness and side effect)	Medication adherence assessments	Medication guidance	Health management (lifestyle)
Ralston JD 2014	√	√	√			√
