# Supplementary material for: Evaluating the Impact of the COVID-19 Pandemic on Telepharmaceutical Service Effectiveness: Systematic Review and Meta-Analysis
Source: J Med Internet Res. 2025 Jul 2;27:e64073. doi: 10.2196/64073 (PMC12268221; doi:10.2196/64073)
Supplement: Multimedia Appendix 10 [file jmir_v27i1e64073_app10.pdf]

# Multimedia Appendix 10: Funnel plots

## 10.1 Medication adherence

*Dichotomous data*

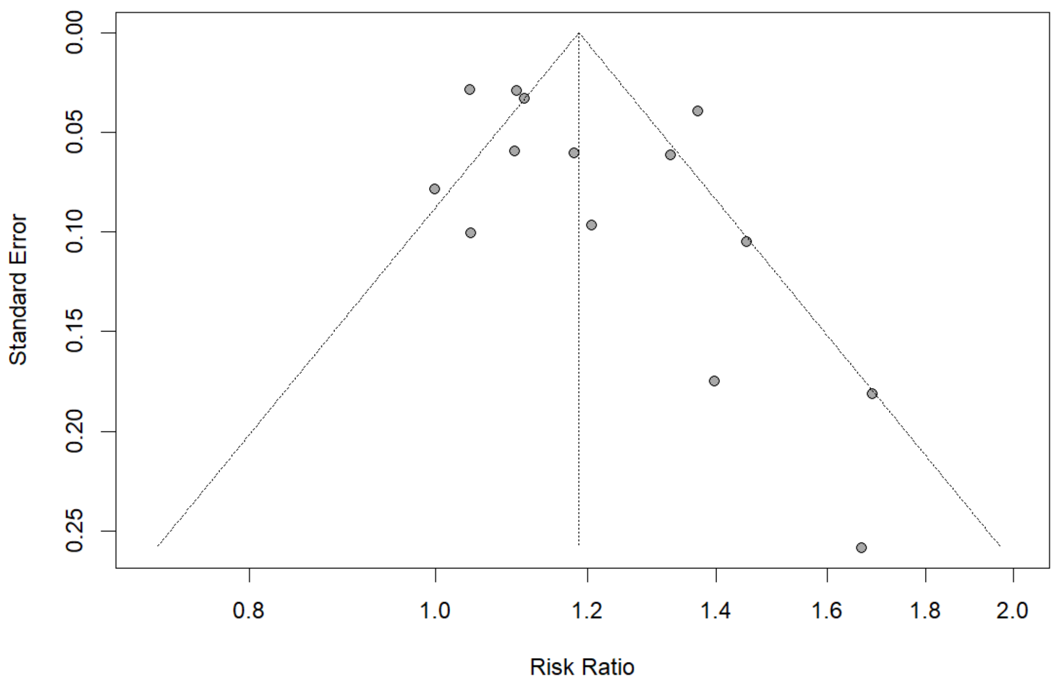


Test result: $t = 1.77$, $df = 12$, $P$ value = 0.10

Bias estimate: 1.7624 ($SE = 0.9955$)

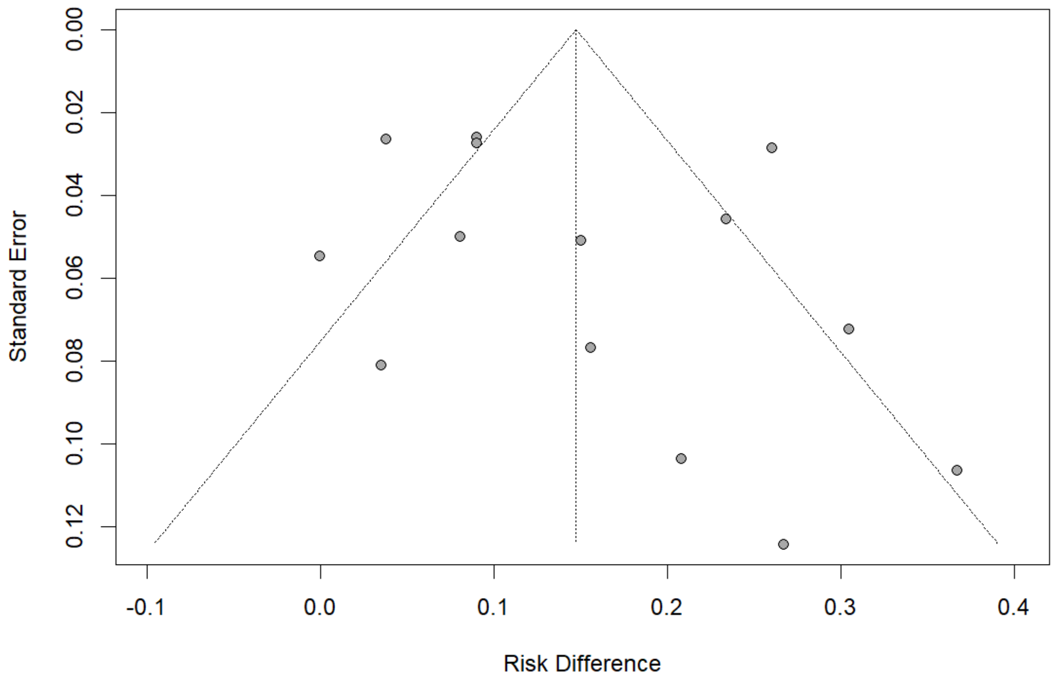


Test result: $t = 1.10$, $df = 12$, $P$ value = 0.29

Bias estimate: 1.4026 ($SE = 1.2711$)

**10.2 Adverse events**

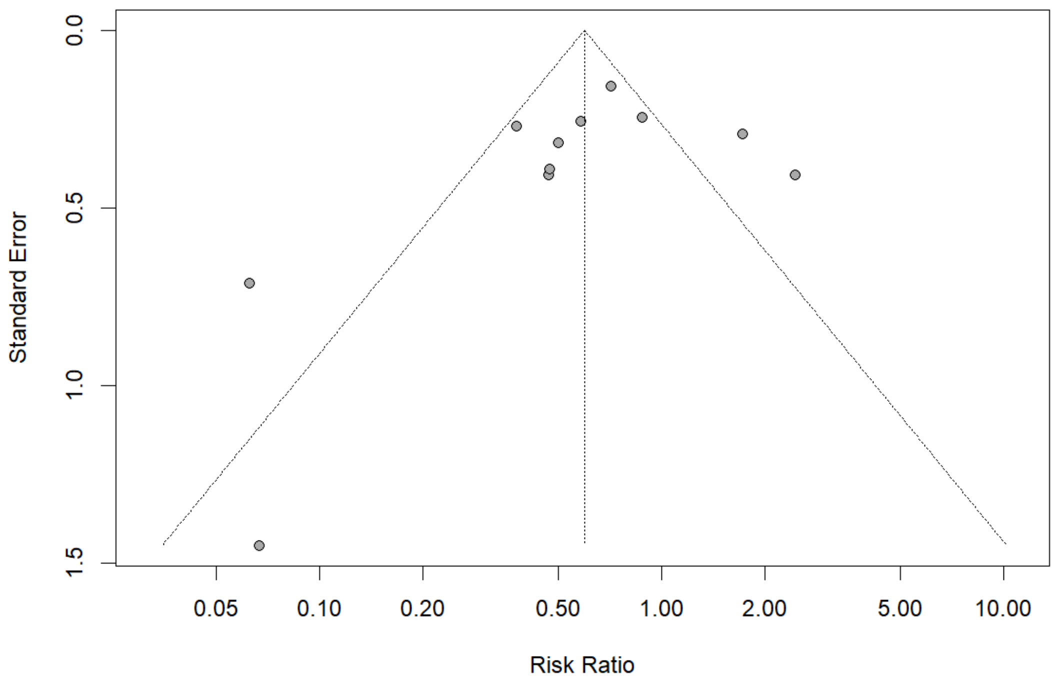


Test result: $t$ = -1.09, $df$ = 9, $P$ value = 0.30

Bias estimate: -1.6181 ($SE$ = 1.4855)

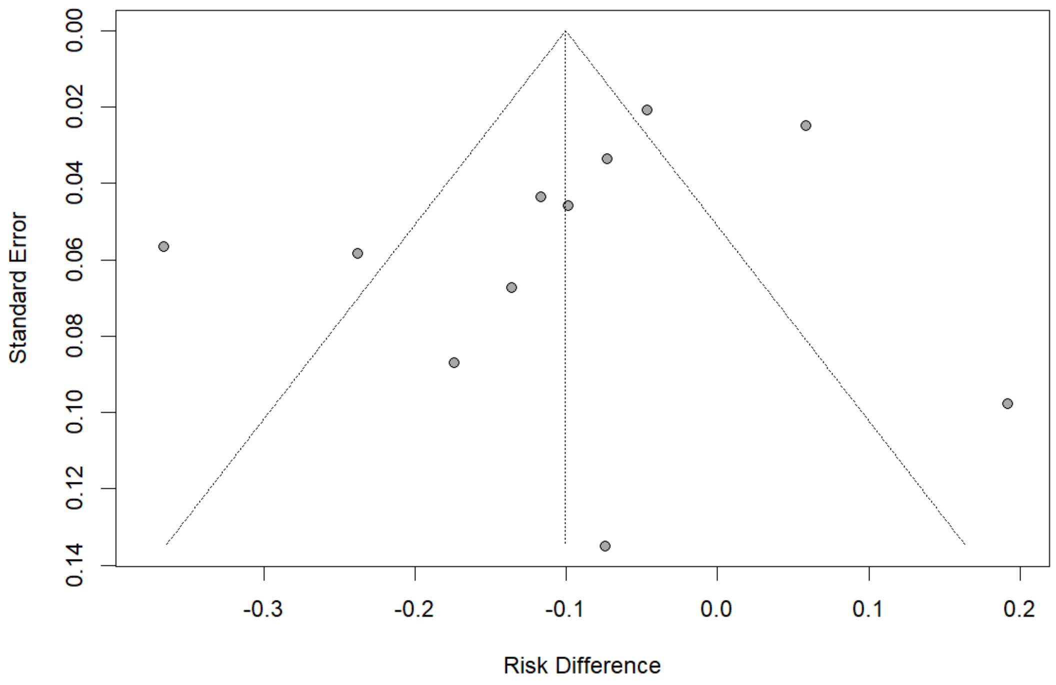


Test result: $t$ = -1.39, $df$ = 9, $P$ value = 0.20

Bias estimate: -2.2512 ($SE$ = 1.6147)
