# Supplementary material for: Evaluating the Impact of the COVID-19 Pandemic on Telepharmaceutical Service Effectiveness: Systematic Review and Meta-Analysis
Source: J Med Internet Res. 2025 Jul 2;27:e64073. doi: 10.2196/64073 (PMC12268221; doi:10.2196/64073)
Supplement: Multimedia Appendix 11 [file jmir_v27i1e64073_app11.pdf]

Multimedia Appendix 11: Forest plots for meta-analysis

11.1 Medication adherence

Dichotomous data

Study	Experimental Events	Experimental Total	Control Events	Control Total	RR	95%-CI	Weight
Before the outbreak							
Alsabbagh MW 2012	38	46	38	48	1.04	[0.86; 1.27]	6.0%
Chen Y 2017	194	200	176	200	1.10	[1.04; 1.17]	10.6%
Elliott RA 2008	77	87	95	118	1.10	[0.98; 1.24]	8.6%
Feng H 2021	27	30	16	30	1.69	[1.18; 2.41]	2.9%
Lyons I 2016	304	340	271	337	1.11	[1.04; 1.19]	10.4%
Magid DJ 2011	96	138	101	145	1.00	[0.86; 1.16]	7.3%
Ralston JD 2014	176	186	179	197	1.04	[0.98; 1.10]	10.6%
Young HN 2012	30	41	22	42	1.40	[0.99; 1.97]	3.0%
Zhang XS 2019	114	120	86	120	1.33	[1.18; 1.49]	8.5%
Random effects model		1188		1237	1.12	[1.05; 1.20]	67.9%
Heterogeneity: $I^2 = 64\%$, $\tau^2 = 0.0053$, $p < 0.01$							
After the outbreak							
Liao QQ 2023	59	60	50	60	1.18	[1.05; 1.33]	8.6%
Liu Y 2022	45	46	31	46	1.45	[1.18; 1.78]	5.7%
Wang ZM(2) 2023	289	300	211	300	1.37	[1.27; 1.48]	10.0%
Ye QM 2022	20	30	12	30	1.67	[1.00; 2.76]	1.6%
Zhang W 2022	41	45	34	45	1.21	[1.00; 1.46]	6.2%
Random effects model		481		481	1.31	[1.19; 1.43]	32.1%
Heterogeneity: $I^2 = 42\%$, $\tau^2 = 0.0042$, $p = 0.14$							
**Random effects model**		**1669**		**1718**	**1.19**	**[1.11; 1.27]**	**100.0%**
Heterogeneity: $I^2 = 78\%$, $\tau^2 = 0.0110$, $p < 0.01$							
Test for subgroup differences: $\chi^2_1 = 6.80$, df = 1 ($p < 0.01$)							

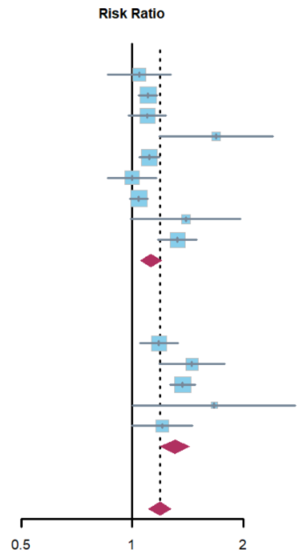

Study	Experimental Events	Experimental Total	Control Events	Control Total	RD	95%-CI	Weight
Before the outbreak							
Alsabbagh MW 2012	38	46	38	48	0.03	[-0.12; 0.19]	5.7%
Chen Y 2017	194	200	176	200	0.09	[0.04; 0.14]	9.7%
Elliott RA 2008	77	87	95	118	0.08	[-0.02; 0.18]	7.9%
Feng H 2021	27	30	16	30	0.37	[0.16; 0.57]	4.2%
Lyons I 2016	304	340	271	337	0.09	[0.04; 0.14]	9.6%
Magid DJ 2011	96	138	101	145	-0.00	[-0.11; 0.11]	7.6%
Ralston JD 2014	176	186	179	197	0.04	[-0.01; 0.09]	9.7%
Young HN 2012	30	41	22	42	0.21	[0.00; 0.41]	4.4%
Zhang XS 2019	114	120	86	120	0.23	[0.14; 0.32]	8.3%
Random effects model		1188		1237	0.11	[0.05; 0.16]	67.0%
Heterogeneity: $I^2 = 68\%$, $\tau^2 = 0.0052$, $p < 0.01$							
After the outbreak							
Liao QQ 2023	59	60	50	60	0.15	[0.05; 0.25]	7.9%
Liu Y 2022	45	46	31	46	0.30	[0.16; 0.45]	6.2%
Wang ZM(2) 2023	289	300	211	300	0.26	[0.20; 0.32]	9.5%
Ye QM 2022	20	30	12	30	0.27	[0.02; 0.51]	3.5%
Zhang W 2022	41	45	34	45	0.16	[0.00; 0.31]	5.9%
Random effects model		481		481	0.23	[0.16; 0.29]	33.0%
Heterogeneity: $I^2 = 29\%$, $\tau^2 = 0.0018$, $p = 0.23$							
**Random effects model**		**1669**		**1718**	**0.15**	**[0.09; 0.20]**	**100.0%**
Heterogeneity: $I^2 = 80\%$, $\tau^2 = 0.0076$, $p < 0.01$							
Test for subgroup differences: $\chi^2_1 = 7.37$, df = 1 ($p < 0.01$)							

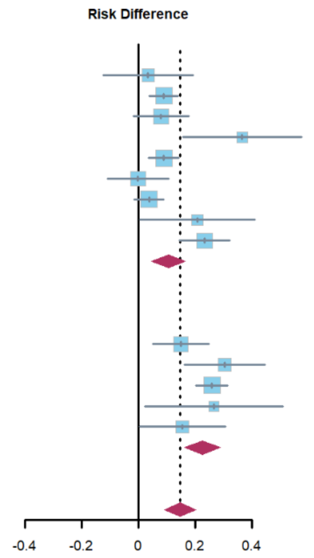

Continuous data

Study	Experimental Total	Experimental Mean	Experimental SD	Control Total	Control Mean	Control SD	MD	95%-CI	Weight
**Before the outbreak**									
Choudhry KN 2018	2038	46.20	33.9000	2040	42.10	33.8000	4.10	[2.02; 6.18]	14.6%
Lauffenburger JC 2019	678	81.90	30.1000	684	81.90	31.0000	0.00	[-3.25; 3.25]	14.3%
Lu Y 2017	200	93.50	5.5000	200	68.00	8.0000	25.50	[24.15; 26.85]	14.7%
Zhao JY 2023	82	90.25	4.2500	82	71.00	9.1250	19.25	[17.07; 21.43]	14.6%
Random effects model	2998			3006			12.26	[0.38; 24.14]	58.2%
Heterogeneity: $I^2 = 99\%$, $\tau^2 = 145.5705$, $p < 0.01$									
**After the outbreak**									
Ibrahim OM 2022	115	95.00	22.5000	118	78.75	32.5000	16.25	[9.09; 23.41]	12.6%
Jiang H 2022	40	90.59	2.7400	40	74.18	2.7100	16.41	[15.22; 17.60]	14.7%
Wang ZM(1) 2023	300	83.62	16.3750	300	67.00	16.0000	16.62	[14.03; 19.22]	14.5%
Random effects model	455			458			16.44	[15.37; 17.52]	41.8%
Heterogeneity: $I^2 = 0\%$, $\tau^2 = 0$, $p = 0.99$									
**Random effects model**	**3453**			**3464**			**14.03**	**[7.37; 20.69]**	**100.0%**

Heterogeneity: $I^2 = 99\%$, $\tau^2 = 77.9920$, $p < 0.01$
Test for subgroup differences: $\chi^2_1 = 0.47$, df = 1 ($p = 0.49$)

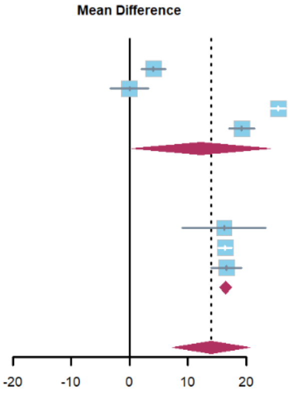

11.2 Medication satisfaction

Dichotomous data

Study	Experimental Events	Experimental Total	Control Events	Control Total	RR	95%-CI	Weight
**Before the outbreak**							
Magid DJ 2013	94	162	69	164	1.38	[1.10; 1.72]	13.6%
Margolis KL 2022	413	618	451	683	1.01	[0.94; 1.09]	19.2%
Xu JY 2023	247	255	171	259	1.47	[1.34; 1.61]	18.8%
Jin X 2021	38	40	30	40	1.27	[1.04; 1.54]	14.9%
Random effects model		1075		1146	1.26	[1.05; 1.51]	66.5%
Heterogeneity: $I^2 = 92\%$, $\tau^2 = 0.0272$, $p < 0.01$							
**After the outbreak**							
Liu Y 2022	43	46	30	46	1.43	[1.15; 1.79]	13.5%
Liao QQ 2023	60	60	58	60	1.03	[0.99; 1.08]	19.9%
Random effects model		106		106	1.19	[0.87; 1.64]	33.5%
Heterogeneity: $I^2 = 87\%$, $\tau^2 = 0.0464$, $p < 0.01$							
**Random effects model**		**1181**		**1252**	**1.23**	**[1.07; 1.42]**	**100.0%**

Heterogeneity: $I^2 = 92\%$, $\tau^2 = 0.0261$, $p < 0.01$
Test for subgroup differences: $\chi^2_1 = 0.08$, df = 1 ($p = 0.77$)

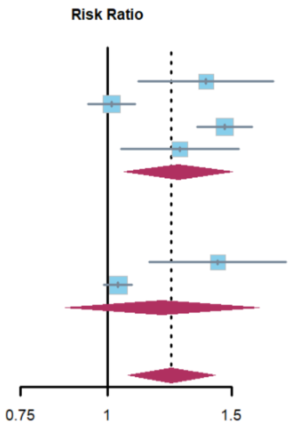

Study	Experimental Events	Experimental Total	Control Events	Control Total	RD	95%-CI	Weight
**Before the outbreak**							
Magid DJ 2013	94	162	69	164	0.16	[0.05; 0.27]	16.3%
Margolis KL 2022	413	618	451	683	0.01	[-0.04; 0.06]	18.7%
Xu JY 2023	247	255	171	259	0.31	[0.25; 0.37]	18.4%
Jin X 2021	38	40	30	40	0.20	[0.05; 0.35]	14.1%
Random effects model		1075		1146	0.17	[0.03; 0.30]	67.5%
Heterogeneity: $I^2 = 95\%$, $\tau^2 = 0.0160$, $p < 0.01$							
**After the outbreak**							
Liu Y 2022	43	46	30	46	0.28	[0.13; 0.44]	13.8%
Liao QQ 2023	60	60	58	60	0.03	[-0.02; 0.09]	18.6%
Random effects model		106		106	0.15	[-0.10; 0.39]	32.5%
Heterogeneity: $I^2 = 89\%$, $\tau^2 = 0.0276$, $p < 0.01$							
**Random effects model**		**1181**		**1252**	**0.16**	**[0.05; 0.26]**	**100.0%**

Heterogeneity: $I^2 = 93\%$, $\tau^2 = 0.0149$, $p < 0.01$
Test for subgroup differences: $\chi^2_1 = 0.02$, df = 1 ($p = 0.89$)

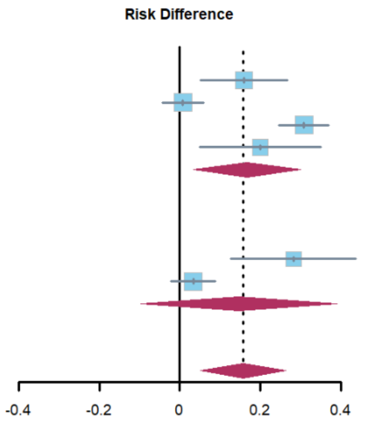

Continuous data

Study	Experimental Total	Experimental Mean	Experimental SD	Control Total	Control Mean	Control SD	MD	95%-CI	Weight
**Before the outbreak**									
Bynum A 2001	15	91.60	9.0000	21	83.00	19.8000	8.60	[-1.02; 18.22]	26.3%
Margolis KL 2013	188	90.00	20.9867	182	88.00	13.7661	2.00	[-1.61; 5.61]	73.7%
Random effects model	203			203			3.73	[-1.96; 9.43]	100.0%
Heterogeneity: $I^2 = 37\%$, $\tau^2 = 8.0537$, $p = 0.21$									
**Random effects model**	**203**			**203**			**3.73**	**[-1.96; 9.43]**	**100.0%**

Heterogeneity: $I^2 = 37\%$, $\tau^2 = 8.0537$, $p = 0.21$
Test for subgroup differences: $\chi^2_0 = 0.00$, df = 0 (p = NA)

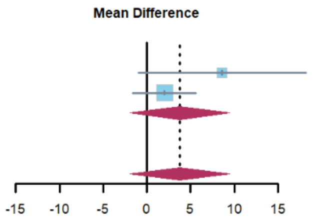

11.3 Adverse events

Study	Experimental Events	Experimental Total	Control Events	Control Total	RR	95%-CI	Weight
**Before the outbreak**							
Li JY 2022	20	204	8	201	2.46	[1.11; 5.46]	9.1%
Shi NN 2021	22	48	12	45	1.72	[0.97; 3.05]	10.3%
Wan JW 2022	15	105	40	105	0.38	[0.22; 0.64]	10.6%
Zhao JY 2023	2	82	32	82	0.06	[0.02; 0.25]	6.1%
Random effects model		439		433	0.60	[0.13; 2.86]	36.1%
Heterogeneity: I^2 = 91%, τ^2 = 2.3246, p < 0.01							
**After the outbreak**							
Liao QQ 2023	0	60	7	60	0.07	[0.00; 1.14]	2.4%
Liu H 2022	14	27	16	27	0.88	[0.54; 1.41]	10.8%
Liu Y 2022	7	46	15	46	0.47	[0.21; 1.04]	9.1%
Wang ZM(1) 2023	14	300	28	300	0.50	[0.27; 0.93]	10.1%
Wang ZM(2) 2023	54	300	76	300	0.71	[0.52; 0.97]	11.5%
Yu JK 2023	20	146	33	140	0.58	[0.35; 0.96]	10.7%
Zhang YL 2023	8	66	17	66	0.47	[0.22; 1.01]	9.3%
Random effects model		945		939	0.64	[0.53; 0.79]	63.9%
Heterogeneity: I^2 = 7%, τ^2 = < 0.0001, p = 0.37							
**Random effects model**		**1384**		**1372**	**0.59**	**[0.36; 0.97]**	**100.0%**
Heterogeneity: I^2 = 77%, τ^2 = 0.5171, p < 0.01							
Test for subgroup differences: χ^2_1 = 0.01, df = 1 (p = 0.94)							

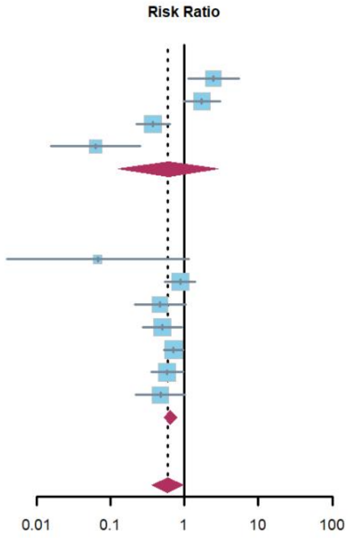

Study	Experimental Events	Experimental Total	Control Events	Control Total	RD	95%-CI	Weight
**Before the outbreak**							
Li JY 2022	20	204	8	201	0.06	[0.01; 0.11]	10.9%
Shi NN 2021	22	48	12	45	0.19	[0.00; 0.38]	7.0%
Wan JW 2022	15	105	40	105	-0.24	[-0.35; -0.12]	9.3%
Zhao JY 2023	2	82	32	82	-0.37	[-0.48; -0.26]	9.4%
Random effects model		439		433	-0.09	[-0.34; 0.15]	36.6%
Heterogeneity: I^2 = 95%, τ^2 = 0.0601, p < 0.01							
**After the outbreak**							
Liao QQ 2023	0	60	7	60	-0.12	[-0.20; -0.03]	10.1%
Liu H 2022	14	27	16	27	-0.07	[-0.34; 0.19]	5.2%
Liu Y 2022	7	46	15	46	-0.17	[-0.34; -0.00]	7.6%
Wang ZM(1) 2023	14	300	28	300	-0.05	[-0.09; -0.01]	11.0%
Wang ZM(2) 2023	54	300	76	300	-0.07	[-0.14; -0.01]	10.6%
Yu JK 2023	20	146	33	140	-0.10	[-0.19; -0.01]	10.0%
Zhang YL 2023	8	66	17	66	-0.14	[-0.27; -0.00]	8.8%
Random effects model		945		939	-0.08	[-0.12; -0.05]	63.4%
Heterogeneity: I^2 = 0%, τ^2 = 0.0004, p = 0.52							
**Random effects model**		**1384**		**1372**	**-0.10**	**[-0.18; -0.02]**	**100.0%**
Heterogeneity: I^2 = 86%, τ^2 = 0.0156, p < 0.01							
Test for subgroup differences: χ^2_1 = 0.01, df = 1 (p = 0.92)							

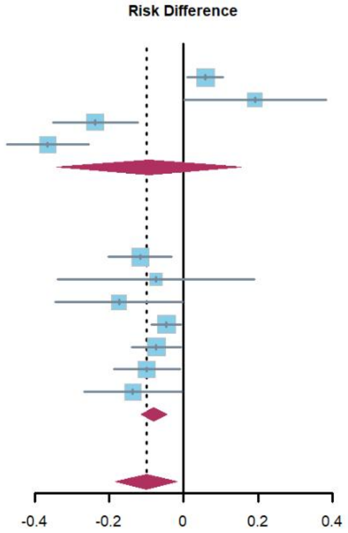

11.4 Diabetes

HbA$_1$c

Study	Experimental Total	Experimental Mean	Experimental SD	Control Total	Control Mean	Control SD	MD	95%-CI	Weight
**Before the outbreak**									
Lu ZW 2021	60	7.50	0.9600	59	8.17	1.3000	-0.67	[-1.08; -0.26]	27.3%
Peasah SK 2020	39	8.20	1.4000	39	8.20	1.3000	0.00	[-0.60; 0.60]	21.7%
Random effects model	99			98			-0.37	[-1.02; 0.28]	49.0%
Heterogeneity: $I^2 = 69\%$, $\tau^2 = 0.1557$, $p = 0.07$									
**After the outbreak**									
Chen XQ 2022	120	8.34	2.4300	120	9.56	3.2900	-1.22	[-1.95; -0.49]	18.2%
Khan YH 2022	54	7.01	0.4878	55	7.24	0.6430	-0.23	[-0.44; -0.01]	32.8%
Random effects model	174			175			-0.66	[-1.62; 0.31]	51.0%
Heterogeneity: $I^2 = 85\%$, $\tau^2 = 0.4174$, $p = 0.01$									
**Random effects model**	**273**			**273**			**-0.48**	**[-0.93; -0.03]**	**100.0%**

Heterogeneity: $I^2 = 71\%$, $\tau^2 = 0.1476$, $p = 0.02$
Test for subgroup differences: $\chi^2_1 = 0.23$, df = 1 ($p = 0.63$)

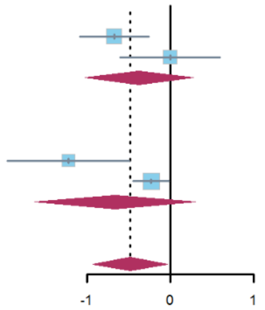

FBG

Study	Experimental Total	Experimental Mean	Experimental SD	Control Total	Control Mean	Control SD	MD	95%-CI	Weight
**Before the outbreak**									
Lu ZW 2021	60	7.31	0.8400	59	7.64	1.1300	-0.33	[-0.69; 0.03]	50.5%
**After the outbreak**									
Chen XQ 2022	120	7.33	1.5100	120	9.26	2.2400	-1.93	[-2.41; -1.45]	49.5%
**Random effects model**	**180**			**179**			**-1.12**	**[-2.69; 0.45]**	**100.0%**

Heterogeneity: $I^2 = 96\%$, $\tau^2 = 1.2329$, $p < 0.01$
Test for subgroup differences: $\chi^2_1 = 27.17$, df = 1 ($p < 0.01$)

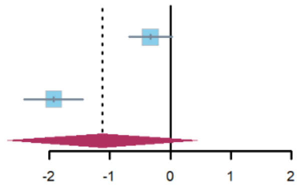

2h PG

Study	Experimental Total	Experimental Mean	Experimental SD	Control Total	Control Mean	Control SD	MD	95%-CI
**After the outbreak**								
Chen XQ 2022	120	7.94	2.2300	120	10.71	3.1900	-2.77	[-3.47; -2.07]

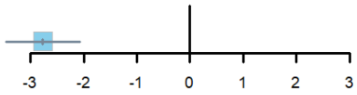

11.5 Hypertension

SBP

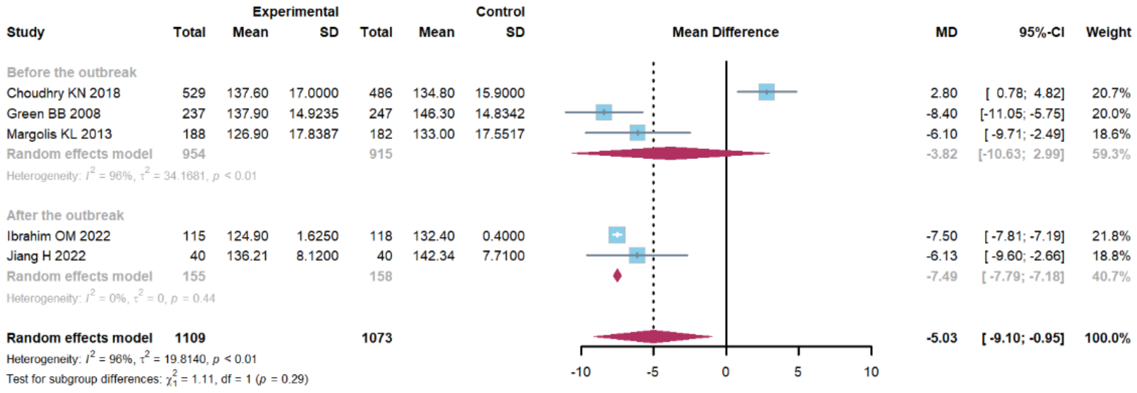

DBP

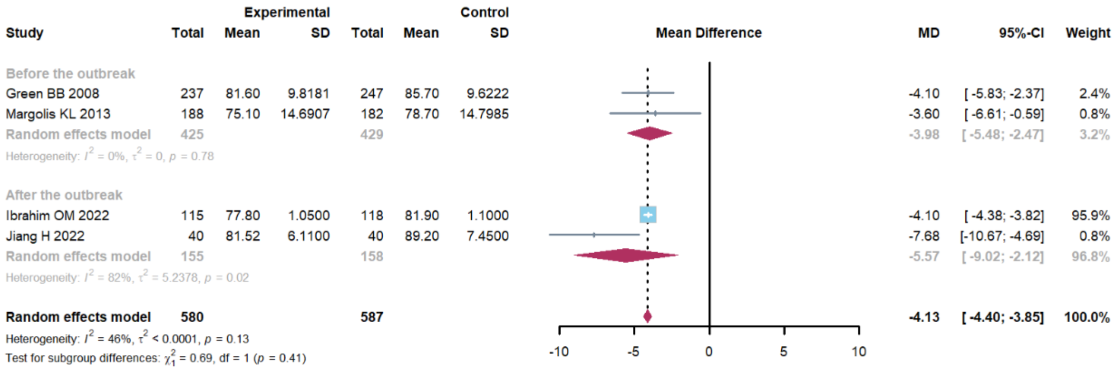

11.6 Anticoagulation

INR (2~3)

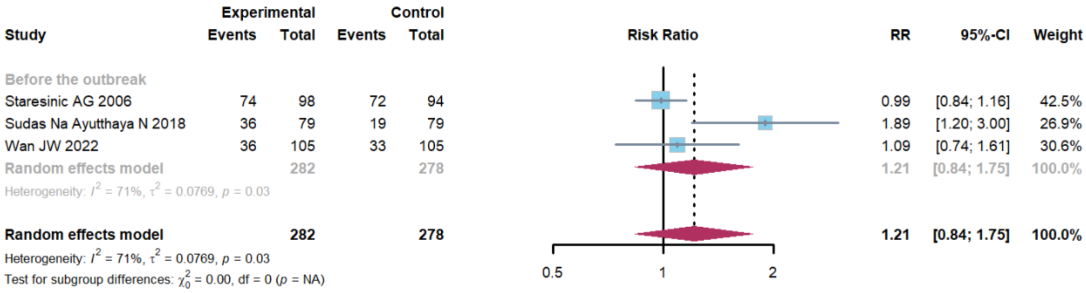

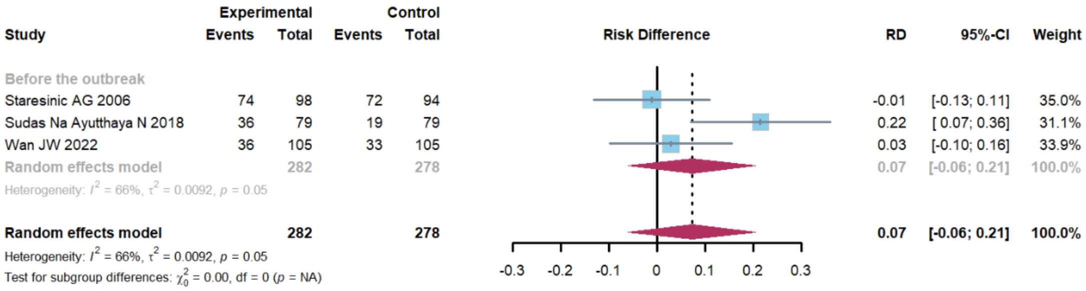

TTR

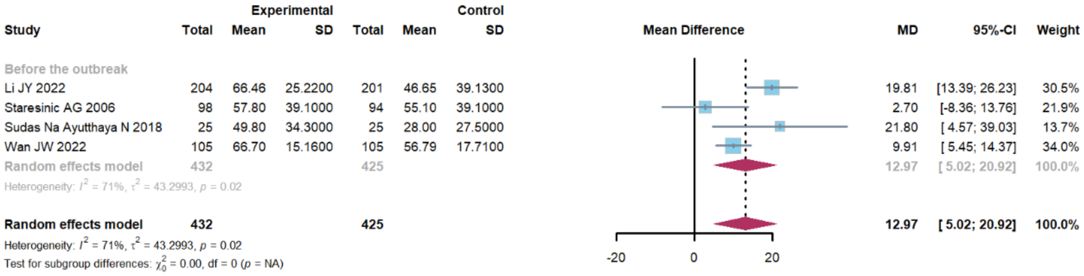

11.7 Other diseases

Cancer (Pain)

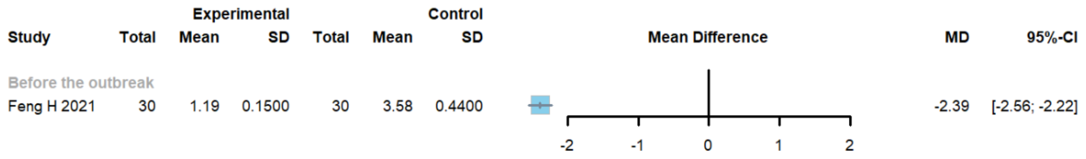

Stroke (Recurrence)

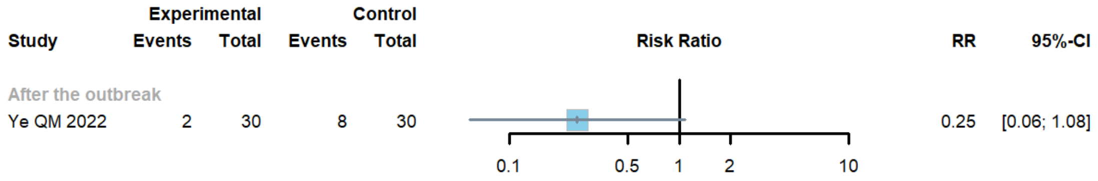

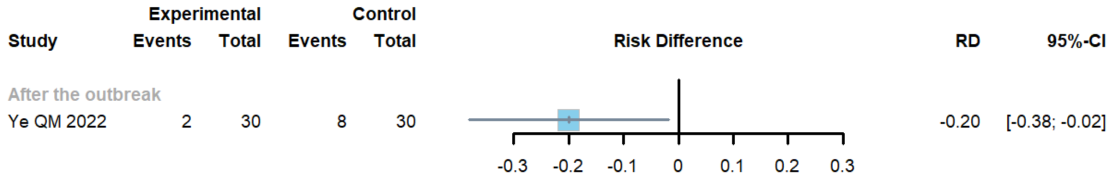

Stroke (BI)

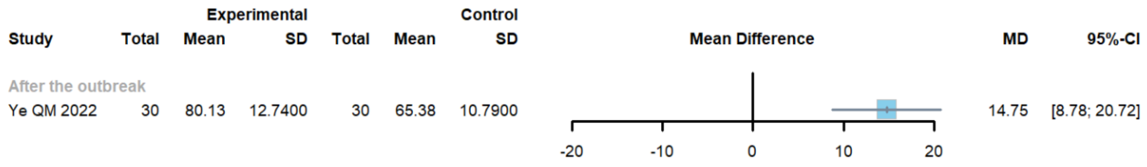

Respiratory diseases (FEV$_1$)

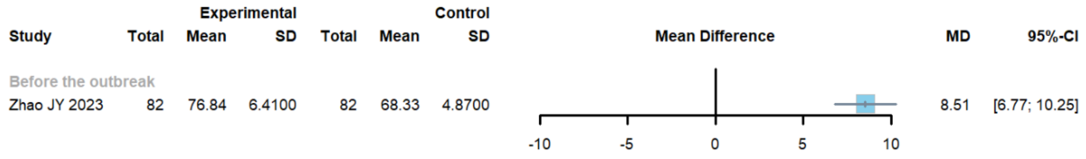

Respiratory diseases (PEF)

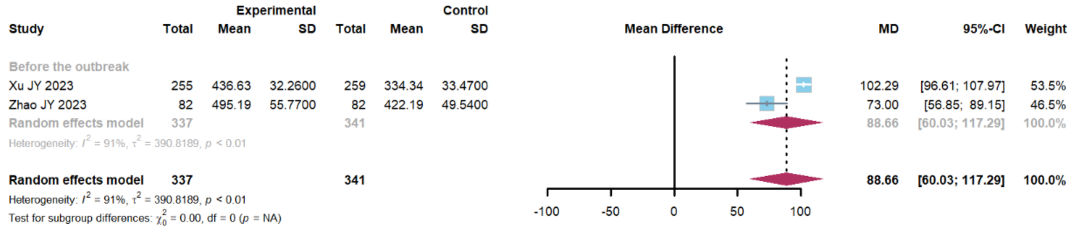

Respiratory diseases (Respiratory function)

Study	Experimental Total	Experimental Mean	Experimental SD	Control Total	Control Mean	Control SD	Mean Difference	MD	95%-CI
Before the outbreak									
Xu JY 2023	255	1.90	0.0300	259	2.59	0.2300	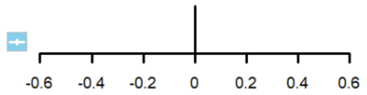	-0.69	[-0.72; -0.66]
