# Supplementary material for: Evaluating the Impact of the COVID-19 Pandemic on Telepharmaceutical Service Effectiveness: Systematic Review and Meta-Analysis
Source: J Med Internet Res. 2025 Jul 2;27:e64073. doi: 10.2196/64073 (PMC12268221; doi:10.2196/64073)
Supplement: Multimedia Appendix 12 [file jmir_v27i1e64073_app12.pdf]

Multimedia Appendix 12: Bubble plots for meta-regression

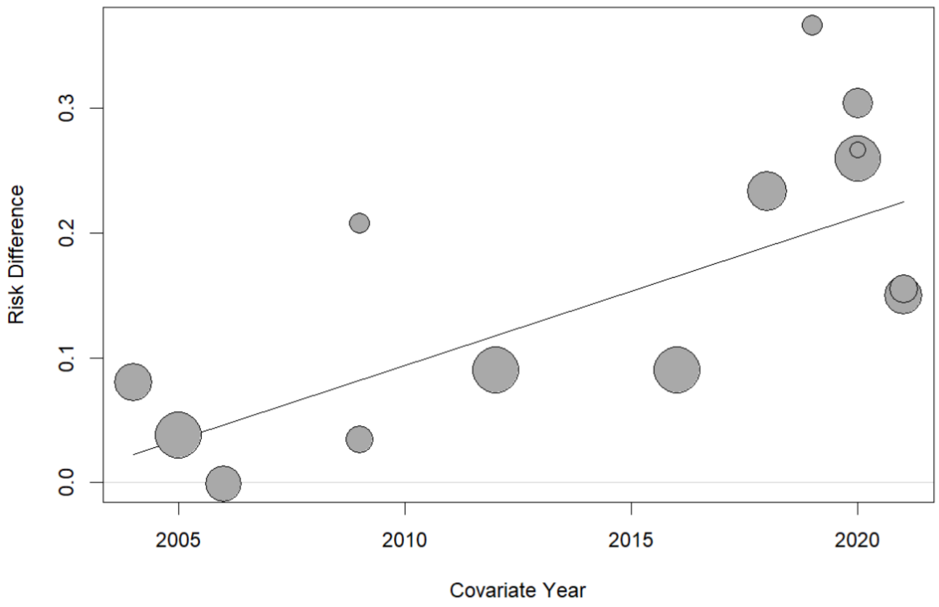

Medication adherence (Dichotomous data & risk difference)

Coefficient & 95% confidence interval: 0.0119 (0.0062 to 0.0176)

Z value: 4.1033

P value: $P < .001$
