# Supplementary material for: Evaluating the Impact of the COVID-19 Pandemic on Telepharmaceutical Service Effectiveness: Systematic Review and Meta-Analysis
Source: J Med Internet Res. 2025 Jul 2;27:e64073. doi: 10.2196/64073 (PMC12268221; doi:10.2196/64073)
Supplement: Multimedia Appendix 13 [file jmir_v27i1e64073_app13.pdf]

Multimedia Appendix 13: Subgroup analysis for TPS by intervention type

<u>Forest Plots</u>

13.1 Medication adherence

Dichotomous data

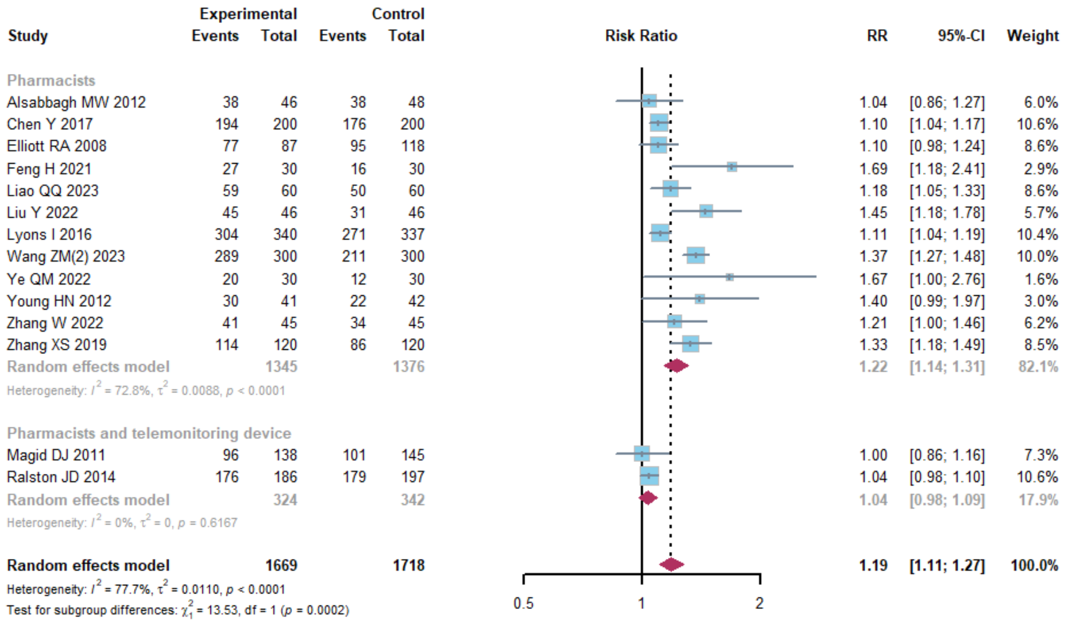

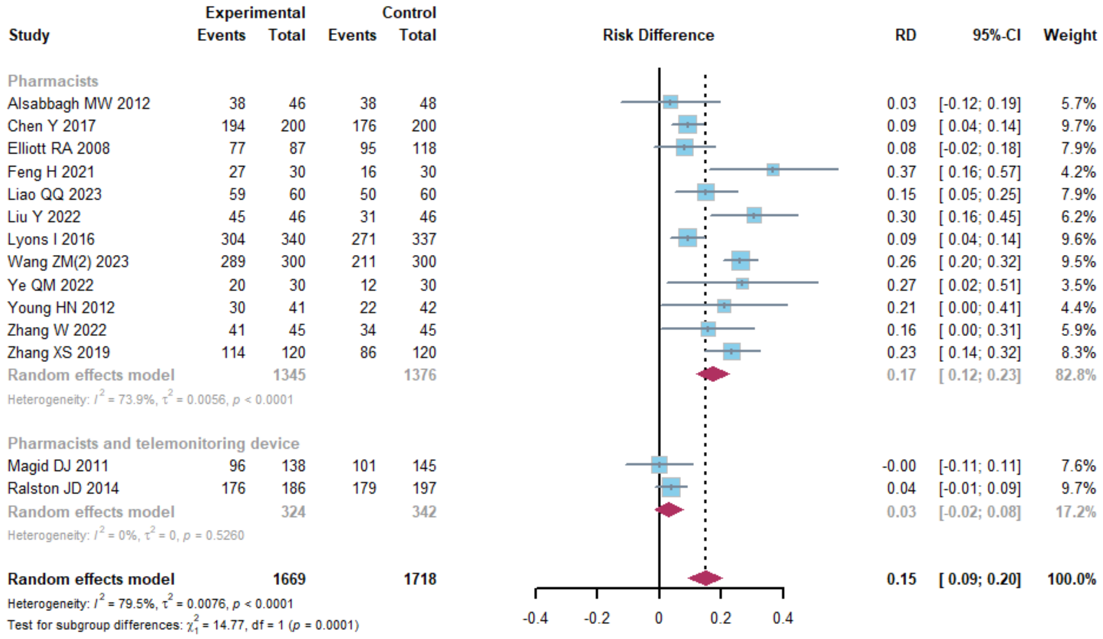

Continuous data

Study	Experimental Total	Experimental Mean	Experimental SD	Control Total	Control Mean	Control SD	MD	95%-CI	Weight
Pharmacists									
Choudhry KN 2018	2038	46.20	33.9000	2040	42.10	33.8000	4.10	[2.02; 6.18]	14.6%
Ibrahim OM 2022	115	95.00	22.5000	118	78.75	32.5000	16.25	[9.09; 23.41]	12.6%
Jiang H 2022	40	90.59	2.7400	40	74.18	2.7100	16.41	[15.22; 17.60]	14.7%
Lauffenburger JC 2019	678	81.90	30.1000	684	81.90	31.0000	0.00	[-3.25; 3.25]	14.3%
Lu Y 2017	200	93.50	5.5000	200	68.00	8.0000	25.50	[24.15; 26.85]	14.7%
Wang ZM(1) 2023	300	83.62	16.3750	300	67.00	16.0000	16.62	[14.03; 19.22]	14.5%
Zhao JY 2023	82	90.25	4.2500	82	71.00	9.1250	19.25	[17.07; 21.43]	14.6%
Random effects model	3453			3464			14.03	[7.37; 20.69]	100.0%
Heterogeneity: $I^2 = 98.5\%$, $\tau^2 = 77.9920$, $p < 0.0001$									
**Random effects model**	**3453**			**3464**			**14.03**	**[7.37; 20.69]**	**100.0%**

Heterogeneity: $I^2 = 98.5\%$, $\tau^2 = 77.9920$, $p < 0.0001$

Test for subgroup differences: $\chi^2_0 = 0.00$, df = 0 (p = NA)

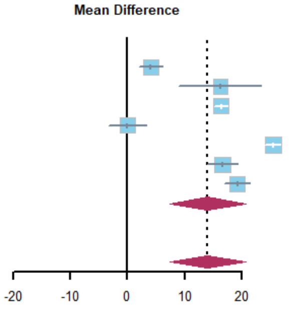

13.2 Medication satisfaction

Dichotomous data

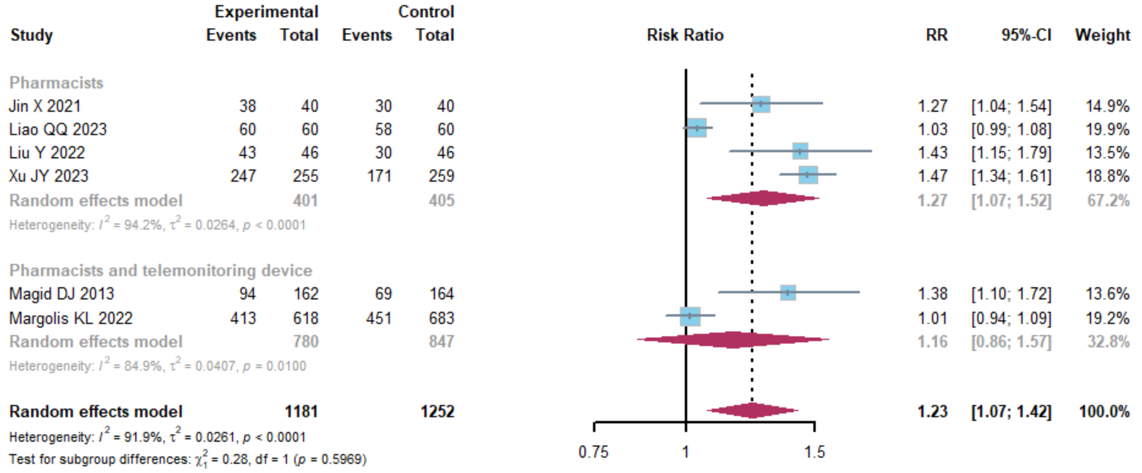

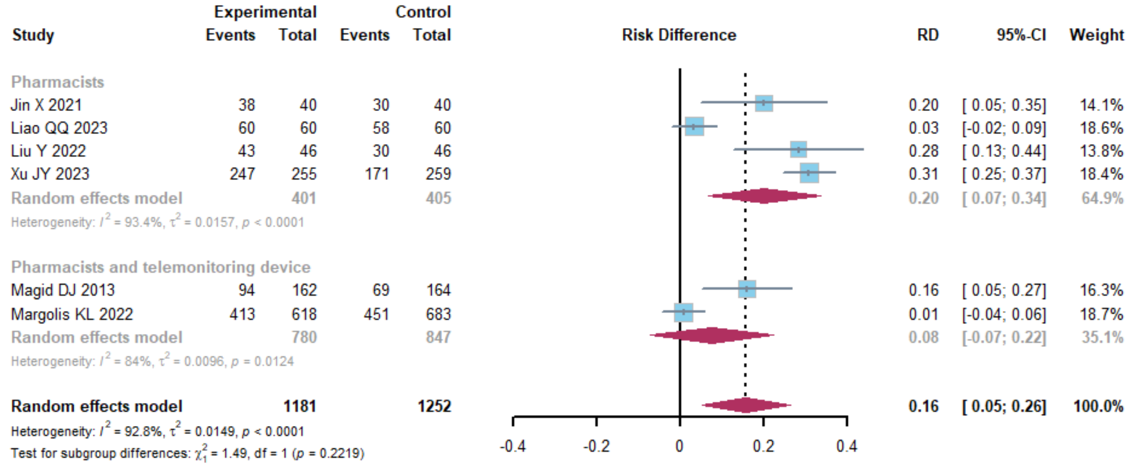

Continuous data

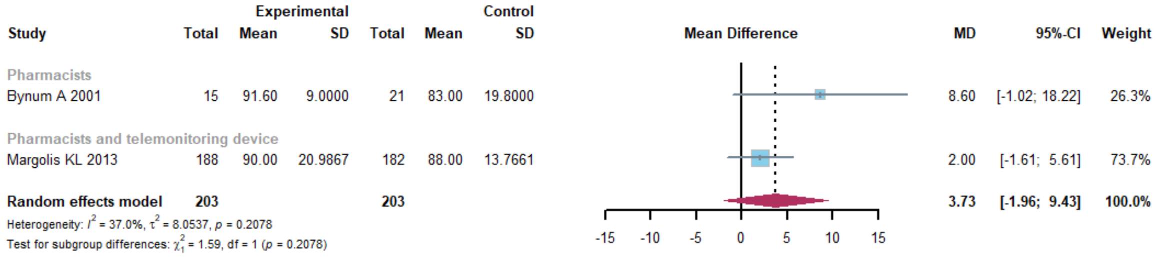

13.3 Adverse events

Study	Experimental Events	Experimental Total	Control Events	Control Total	RR	95%-CI	Weight
Pharmacists							
Li JY 2022	20	204	8	201	2.46	[1.11; 5.46]	9.1%
Liao QQ 2023	0	60	7	60	0.07	[0.00; 1.14]	2.4%
Liu H 2022	14	27	16	27	0.88	[0.54; 1.41]	10.8%
Liu Y 2022	7	46	15	46	0.47	[0.21; 1.04]	9.1%
Shi NN 2021	22	48	12	45	1.72	[0.97; 3.05]	10.3%
Wan JW 2022	15	105	40	105	0.38	[0.22; 0.64]	10.6%
Wang ZM(1) 2023	14	300	28	300	0.50	[0.27; 0.93]	10.1%
Wang ZM(2) 2023	54	300	76	300	0.71	[0.52; 0.97]	11.5%
Yu JK 2023	20	146	33	140	0.58	[0.35; 0.96]	10.7%
Zhang YL 2023	8	66	17	66	0.47	[0.22; 1.01]	9.3%
Zhao JY 2023	2	82	32	82	0.06	[0.02; 0.25]	6.1%
Random effects model		1384		1372	0.59	[0.36; 0.97]	100.0%
Heterogeneity: $I^2 = 76.7\%$, $\tau^2 = 0.5171$, $p < 0.0001$							
**Random effects model**		**1384**		**1372**	**0.59**	**[0.36; 0.97]**	**100.0%**

Heterogeneity: $I^2 = 76.7\%$, $\tau^2 = 0.5171$, $p < 0.0001$

Test for subgroup differences: $\chi^2_0 = 0.00$, df = 0 (p = NA)

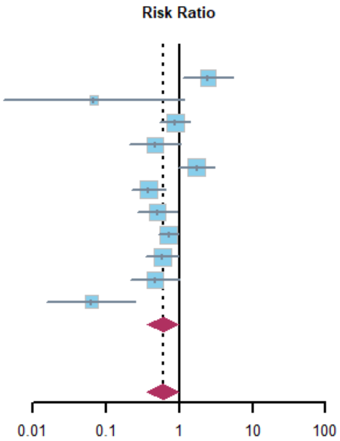

Study	Experimental Events	Experimental Total	Control Events	Control Total	RD	95%-CI	Weight
Pharmacists							
Li JY 2022	20	204	8	201	0.06	[0.01; 0.11]	10.9%
Liao QQ 2023	0	60	7	60	-0.12	[-0.20; -0.03]	10.1%
Liu H 2022	14	27	16	27	-0.07	[-0.34; 0.19]	5.2%
Liu Y 2022	7	46	15	46	-0.17	[-0.34; -0.00]	7.6%
Shi NN 2021	22	48	12	45	0.19	[0.00; 0.38]	7.0%
Wan JW 2022	15	105	40	105	-0.24	[-0.35; -0.12]	9.3%
Wang ZM(1) 2023	14	300	28	300	-0.05	[-0.09; -0.01]	11.0%
Wang ZM(2) 2023	54	300	76	300	-0.07	[-0.14; -0.01]	10.6%
Yu JK 2023	20	146	33	140	-0.10	[-0.19; -0.01]	10.0%
Zhang YL 2023	8	66	17	66	-0.14	[-0.27; -0.00]	8.8%
Zhao JY 2023	2	82	32	82	-0.37	[-0.48; -0.26]	9.4%
Random effects model		1384		1372	-0.10	[-0.18; -0.02]	100.0%
Heterogeneity: $I^2 = 86.4\%$, $\tau^2 = 0.0156$, $p < 0.0001$							
**Random effects model**		**1384**		**1372**	**-0.10**	**[-0.18; -0.02]**	**100.0%**

Heterogeneity: $I^2 = 86.4\%$, $\tau^2 = 0.0156$, $p < 0.0001$

Test for subgroup differences: $\chi^2_0 = 0.00$, df = 0 (p = NA)

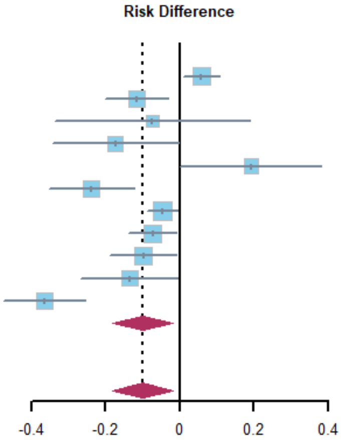

Summary of findings and GRADE certainty of evidence

Outcomes	Subgroup	Study characteristic	Relative effect (RR)	Absolute effect (RD/MD)	Risk of bias	Indirectness	Inconsistency	Imprecision	Other consideration	Rating
Medication adherence (D)	Overall	3387 patients from 14 studies	1.19 (1.11 to 1.27)	0.15 (0.09 to 0.20)	Serious[a]	Not serious	Not serious	Not serious	Not serious	Moderate
	Pharmacists	2721 patients from 12 studies	1.22 (1.14 to 1.31)	0.17 (0.12 to 0.23)	Serious[a]	Not serious	Not serious	Not serious	Not serious	Moderate
	Pharmacists and telemonitoring device	666 patients from 2 studies	1.04 (0.98 to 1.09)	0.03 (-0.02, 0.08)	Serious[a]	Not serious	Not serious	Serious[d]	Not serious	Low
Medication adherence (C)	Overall	6917 patients from 7 studies	N/A	14.03 (7.37 to 20.69)	Serious[a]	Not serious	Not serious	Not serious	Not serious	Moderate
	Pharmacists	6917 patients from 7 studies	N/A	14.03 (7.37 to 20.69)	Serious[a]	Not serious	Not serious	Not serious	Not serious	Moderate
Medication satisfaction (D)	Overall	2433 patients from 6 studies	1.23 (1.07 to 1.42)	0.16 (0.05 to 0.26)	Serious[a]	Not serious	Serious[c]	Not serious	Not serious	Low
	Pharmacists	806 patients from 4 studies	1.27 (1.07 to 1.52)	0.20 (0.07 to 0.34)	Very serious[b]	Not serious	Not serious	Not serious	Not serious	Low
	Pharmacists and telemonitoring device	1627 patients from 2 studies	1.16 (0.86 to 1.57)	0.08 (-0.07 to 0.22)	Serious[a]	Not serious	Serious[c]	Serious[d]	Not serious	Very low
Medication satisfaction (C)	Overall	406 patients from 2 studies	N/A	3.73 (-1.96 to 9.43)	Serious[a]	Not serious	Not serious	Serious[d]	Not serious	Low
	Pharmacists	36 patients from 1 study	N/A	8.60 (-1.02 to 18.22)	Serious[a]	Not serious	Not serious	Serious[d]	Not serious	Low
	Pharmacists and telemonitoring device	370 patients from 1 study	N/A	2.00 (-1.61 to 5.61)	Serious[a]	Not serious	Not serious	Serious[d]	Not serious	Low

Outcomes	Subgroup	Study characteristic	Relative effect (RR)	Absolute effect (RD/MD)	Risk of bias	Indirectness	Inconsistency	Imprecision	Other consideration	Rating
Adverse events (D)	Overall	2756 patients from 11 studies	0.59 (0.36 to 0.97)	-0.10 (-0.18 to -0.02)	Serious[a]	Not serious	Serious[c]	Not serious	Not serious	Low
	Pharmacists	2756 patients from 11 studies	0.59 (0.36 to 0.97)	-0.10 (-0.18 to -0.02)	Serious[a]	Not serious	Serious[c]	Not serious	Not serious	Low

Abbreviations: D (dichotomous outcome); C (continuous outcome); RR (risk ratio); RD (risk difference); MD (mean difference); N/A (Not applicable).

a. Risk of bias: Serious. The risk or bias of almost every study contributing to the outcomes was serious.

b. Risk of bias: Very serious. The risk or bias of almost every study contributing to the outcomes was very serious.

c. Inconsistency: Serious. The effect sizes between studies were not similar and considering heterogeneity with bigger I^2 (>50%).

d. Imprecision: Serious. Downgraded due to wide confidence intervals crossing the null.
