# Supplementary material for: Evaluating the Impact of the COVID-19 Pandemic on Telepharmaceutical Service Effectiveness: Systematic Review and Meta-Analysis
Source: J Med Internet Res. 2025 Jul 2;27:e64073. doi: 10.2196/64073 (PMC12268221; doi:10.2196/64073)
Supplement: Multimedia Appendix 14 [file jmir_v27i1e64073_app14.pdf]

# Multimedia Appendix 14: Subgroup analysis for different regions of TPS

## Forest Plots

### 14.1 Medication adherence

*Dichotomous data*

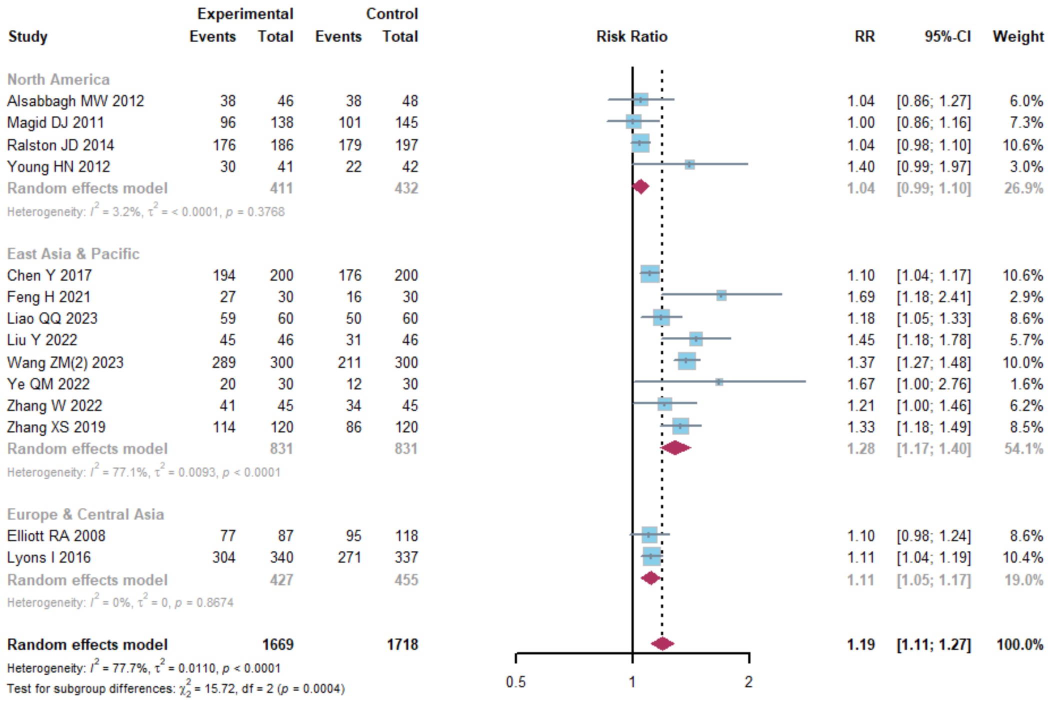


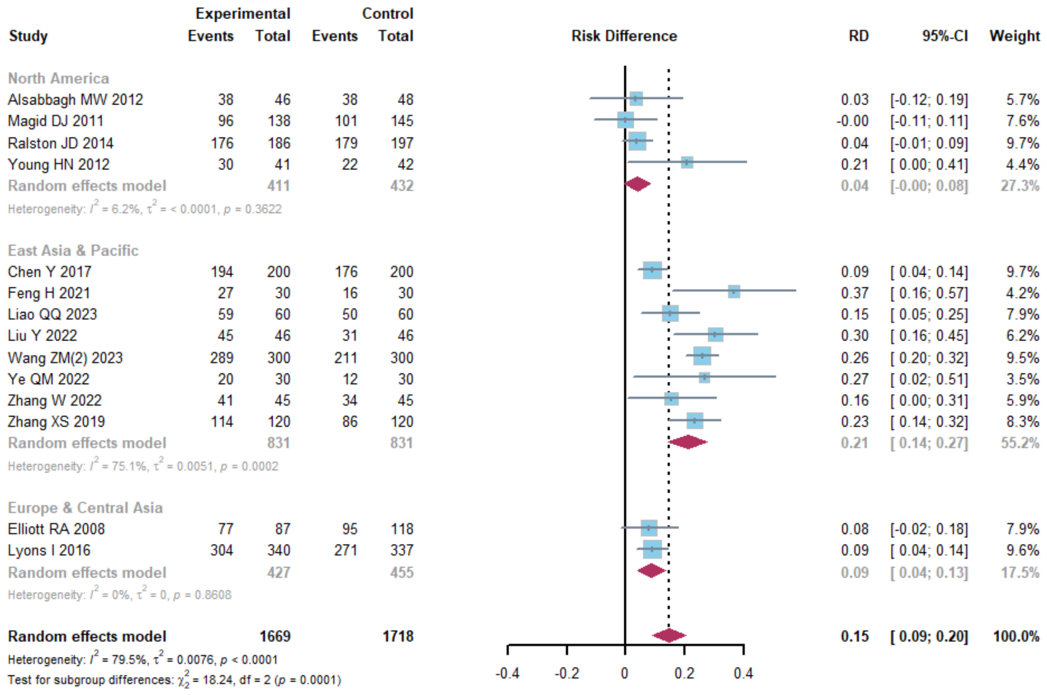

*Continuous data*

| Study | Experimental Total | Experimental Mean | Experimental SD | Control Total | Control Mean | Control SD | MD | 95%-CI | Weight |
|---|---|---|---|---|---|---|---|---|---|
| **North America** | | | | | | | | | |
| Choudhry KN 2018 | 2038 | 46.20 | 33.9000 | 2040 | 42.10 | 33.8000 | 4.10 | [ 2.02; 6.18] | 14.6% |
| Lauffenburger JC 2019 | 678 | 81.90 | 30.1000 | 684 | 81.90 | 31.0000 | 0.00 | [-3.25; 3.25] | 14.3% |
| Random effects model | 2716 | | | 2724 | | | 2.25 | [-1.75; 6.25] | 28.9% |
| Heterogeneity: $I^2 = 77\%$, $\tau^2 = 6.4724$, $p = 0.0370$ | | | | | | | | | |
| **Middle East & North Africa** | | | | | | | | | |
| Ibrahim OM 2022 | 115 | 95.00 | 22.5000 | 118 | 78.75 | 32.5000 | 16.25 | [ 9.09; 23.41] | 12.6% |
| **East Asia & Pacific** | | | | | | | | | |
| Jiang H 2022 | 40 | 90.59 | 2.7400 | 40 | 74.18 | 2.7100 | 16.41 | [15.22; 17.60] | 14.7% |
| Lu Y 2017 | 200 | 93.50 | 5.5000 | 200 | 68.00 | 8.0000 | 25.50 | [24.15; 26.85] | 14.7% |
| Wang ZM(1) 2023 | 300 | 83.62 | 16.3750 | 300 | 67.00 | 16.0000 | 16.62 | [14.03; 19.22] | 14.5% |
| Zhao JY 2023 | 82 | 90.25 | 4.2500 | 82 | 71.00 | 9.1250 | 19.25 | [17.07; 21.43] | 14.6% |
| Random effects model | 622 | | | 622 | | | 19.49 | [15.27; 23.71] | 58.5% |
| Heterogeneity: $I^2 = 97.2\%$, $\tau^2 = 17.6329$, $p < 0.0001$ | | | | | | | | | |
| **Random effects model** | **3453** | | | **3464** | | | **14.03** | **[ 7.37; 20.69]** | **100.0%** |

Heterogeneity: $I^2 = 98.5\%$, $\tau^2 = 77.9920$, $p < 0.0001$

Test for subgroup differences: $\chi^2_2 = 35.97$, df = 2 ($p < 0.0001$)

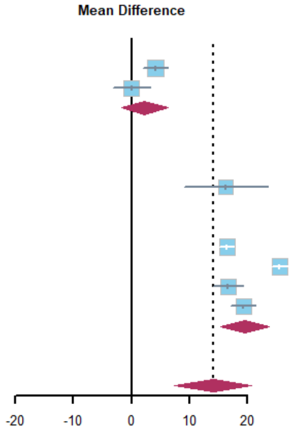

## 14.2 Medication satisfaction

### *Dichotomous data*

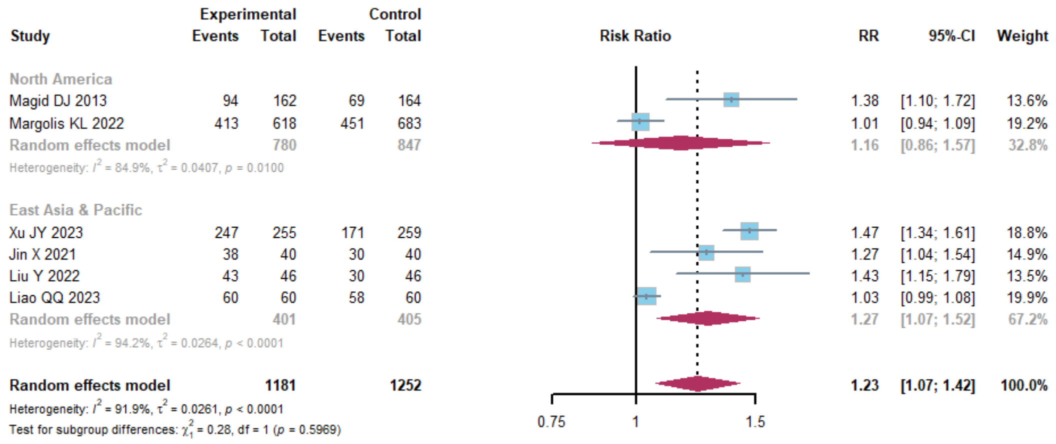


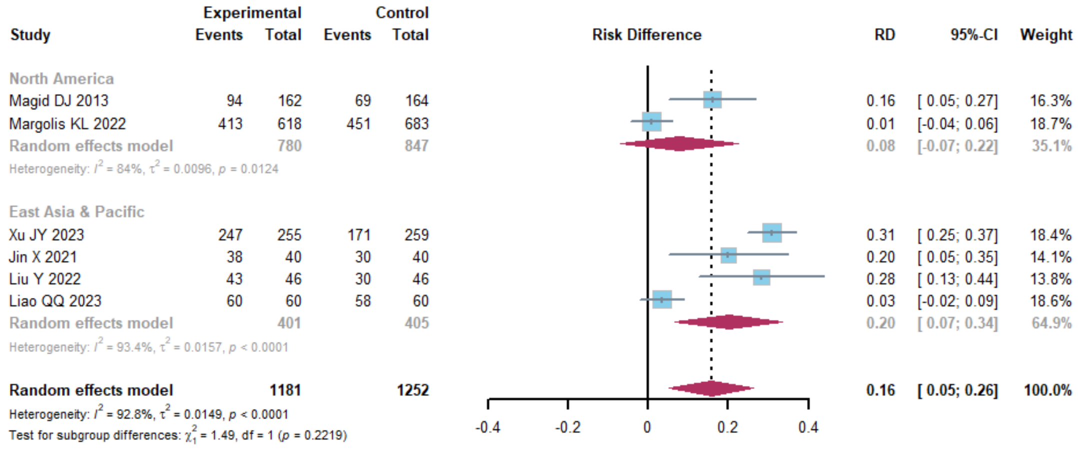


### *Continuous data*

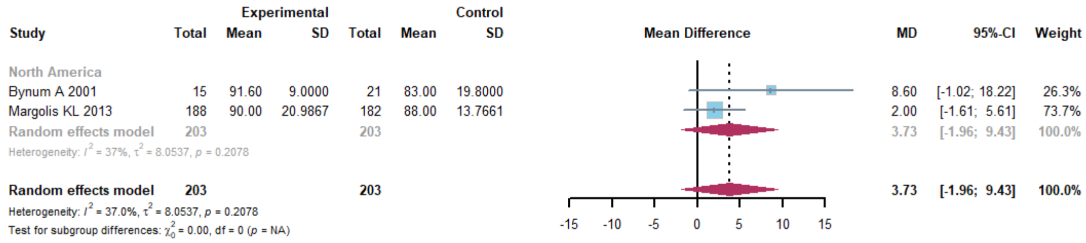

## 14.3 Adverse events

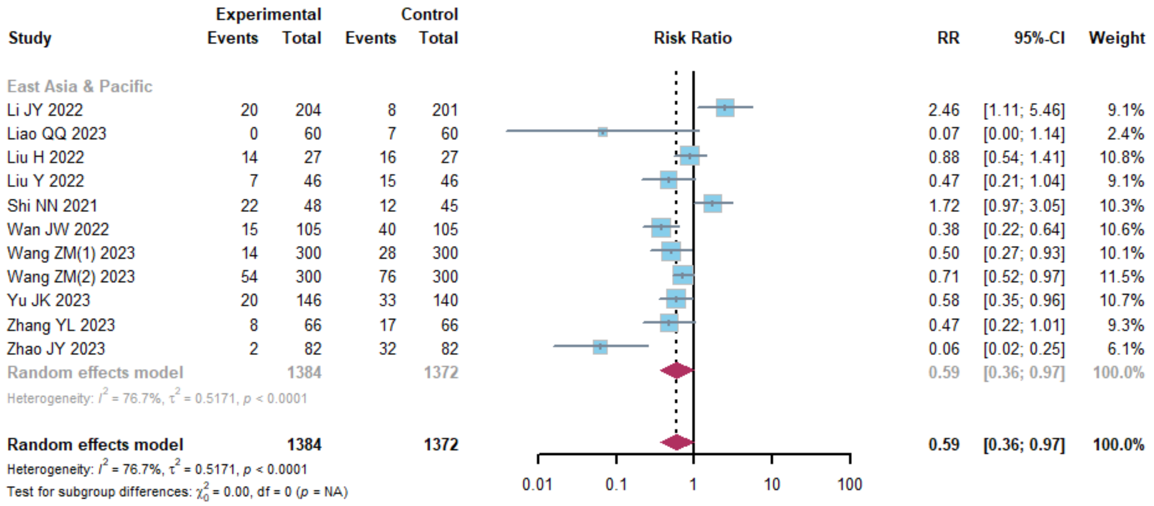


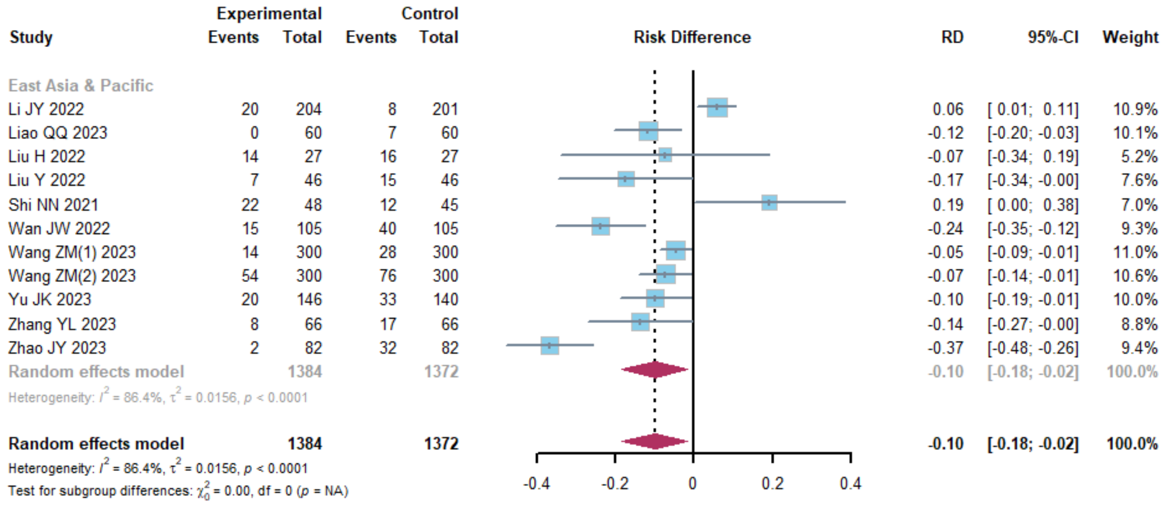

**Summary of findings and GRADE certainty of evidence**

| Outcomes | Subgroup | Study characteristic | Relative effect (RR) | Absolute effect (RD/MD) | Risk of bias | Indirectness | Inconsistency | Imprecision | Other consideration | Rating |
|---|---|---|---|---|---|---|---|---|---|---|
| Medication adherence (D) | Overall | 3387 patients from 14 studies | 1.19 (1.11 to 1.27) | 0.15 (0.09 to 0.20) | Serious[a] | Not serious | Not serious | Not serious | Not serious | Moderate |
| | North America | 843 patients from 4 studies | 1.04 (0.99 to 1.10) | 0.04 (-0.00 to 0.08) | Serious[a] | Not serious | Not serious | Serious[d] | Not serious | Low |
| | East Aisa & Pacific | 1662 patients from 8 studies | 1.28 (1.17 to 1.40) | 0.21 (0.14 to 0.27) | Very serious[b] | Not serious | Not serious | Not serious | Not serious | Low |
| | Europe & Central Asia | 882 patients from 2 studies | 1.11 (1.05 to 1.17) | 0.09 (0.04 to 0.13) | Serious[a] | Not serious | Not serious | Not serious | Not serious | Moderate |
| Medication adherence (C) | Overall | 6917 patients from 7 studies | N/A | 14.03 (7.37 to 20.69) | Serious[a] | Not serious | Not serious | Not serious | Not serious | Moderate |
| | North America | 5440 patients from 2 studies | N/A | 2.25 (-1.75 to 6.25) | Not serious | Not serious | Serious[c] | Serious[d] | Not serious | Low |
| | Middle East & North Africa | 233 patients from 1 study | N/A | 16.25 (9.09 to 23.41) | Very serious[b] | Not serious | Not serious | Very serious[e] | Not serious | Very low |
| | East Asia & Pacific | 1244 patients from 4 studies | N/A | 19.49 (15.27 to 23.71) | Serious[a] | Not serious | Not serious | Not serious | Not serious | Moderate |
| Medication satisfaction (D) | Overall | 2433 patients from 6 studies | 1.23 (1.07 to 1.42) | 0.16 (0.05 to 0.26) | Serious[a] | Not serious | Serious[c] | Not serious | Not serious | Low |
| | North America | 1627 patients from 2 studies | 1.16 (0.86 to 1.57) | 0.08 (-0.07 to 0.22) | Serious[a] | Not serious | Serious[c] | Serious[d] | Not serious | Very low |
| | East Asia & Pacific | 806 patients from | 1.27 | 0.20 | Very | Not serious | Not serious | Not serious | Not serious | Low |

| Outcomes | Subgroup | Study characteristic | Relative effect (RR) | Absolute effect (RD/MD) | Risk of bias | Indirectness | Inconsistency | Imprecision | Other consideration | Rating |
|---|---|---|---|---|---|---|---|---|---|---|
| | | 4 studies | (1.07 to 1.52) | (0.07 to 0.34) | serious[b] | | | | | |
| Medication satisfaction (C) | Overall | 406 patients from 2 studies | N/A | 3.73 (-1.96 to 9.43) | Serious[a] | Not serious | Not serious | Serious[d] | Not serious | Low |
| | North America | 406 patients from 2 studies | N/A | 3.73 (-1.96 to 9.43) | Serious[a] | Not serious | Not serious | Serious[d] | Not serious | Low |
| Adverse events (D) | Overall | 2756 patients from 11 studies | 0.59 (0.36 to 0.97) | -0.10 (-0.18 to -0.02) | Serious[a] | Not serious | Serious[c] | Not serious | Not serious | Low |
| | East Asia & Pacific | 2756 patients from 11 studies | 0.59 (0.36 to 0.97) | -0.10 (-0.18 to -0.02) | Serious[a] | Not serious | Serious[c] | Not serious | Not serious | Low |

Abbreviations: D (dichotomous outcome); C (continuous outcome); RR (risk ratio); RD (risk difference); MD (mean difference); N/A (Not applicable).

a. Risk of bias: Serious. The risk or bias of almost every study contributing to the outcomes was serious.

b. Risk of bias: Very serious. The risk or bias of almost every study contributing to the outcomes was very serious.

c. Inconsistency: Serious. The effect sizes between studies were not similar and considering heterogeneity with bigger $I^2$ (>50%).

d. Imprecision: Serious. Downgraded due to wide confidence intervals crossing the null.

e. Imprecision: Very serious. Downgraded due to sample size which is smaller than 30-50% of the OIS.
