# Supplementary material for: Evaluating the Impact of the COVID-19 Pandemic on Telepharmaceutical Service Effectiveness: Systematic Review and Meta-Analysis
Source: J Med Internet Res. 2025 Jul 2;27:e64073. doi: 10.2196/64073 (PMC12268221; doi:10.2196/64073)

Multimedia Appendix 15: Sensitivity analyses

15.1 Medication adherence

Dichotomous data

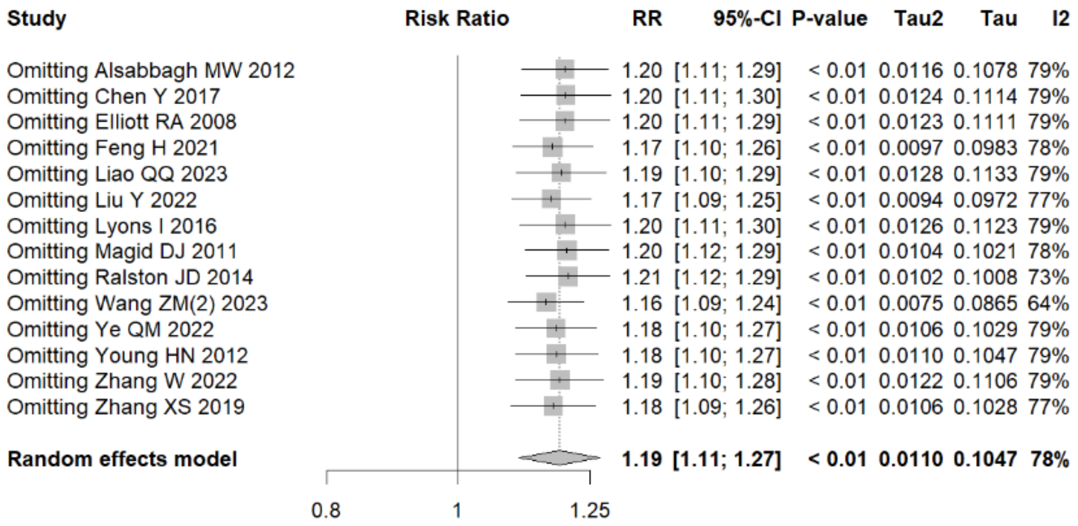

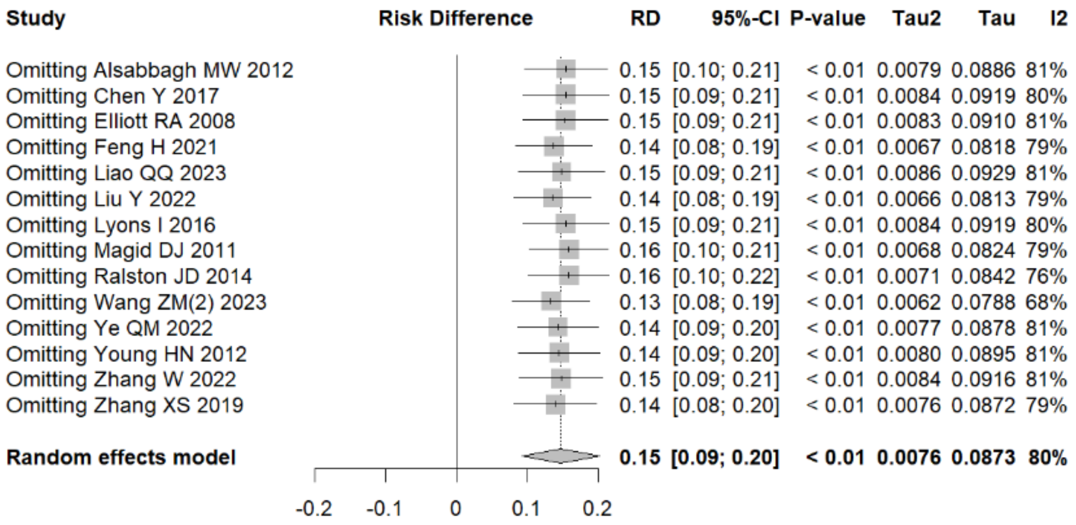

Continuous data

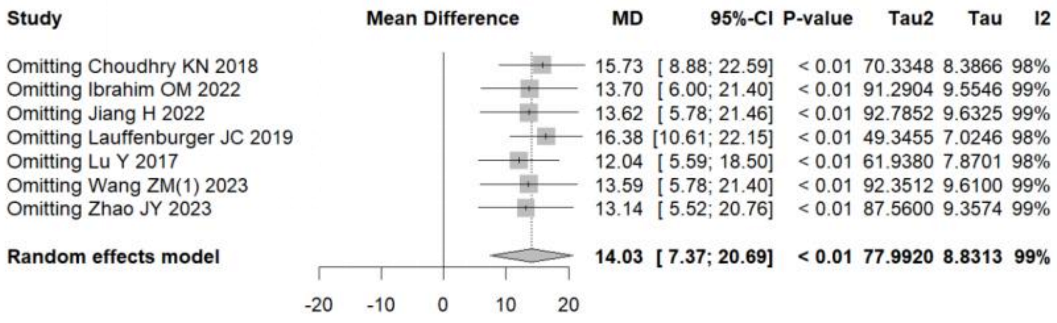

15.2 Medication satisfaction

Dichotomous data

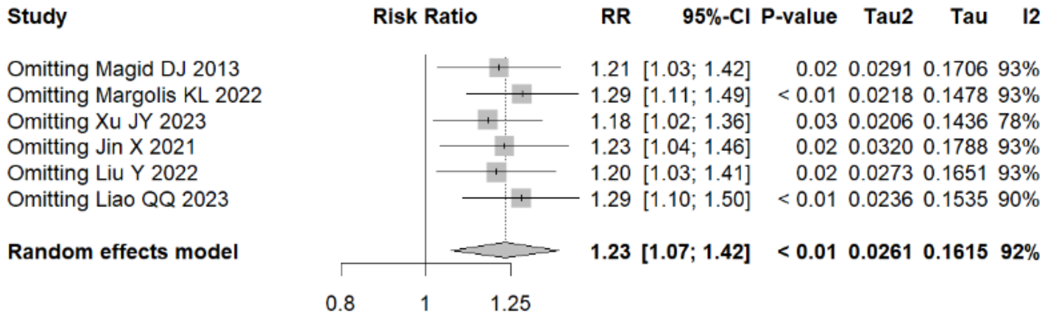

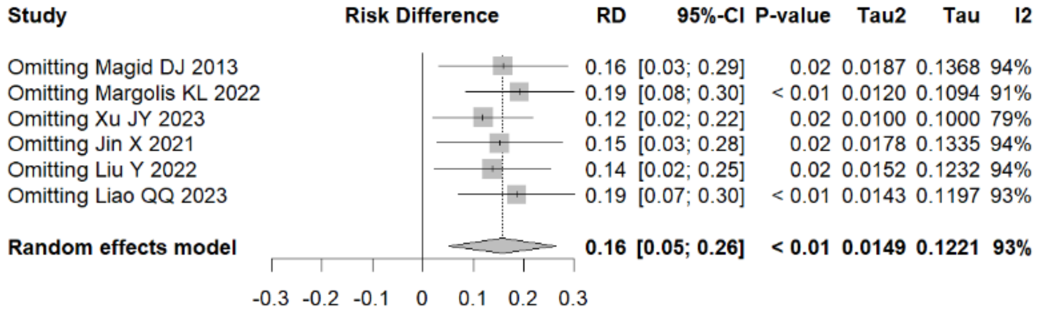

Continuous data

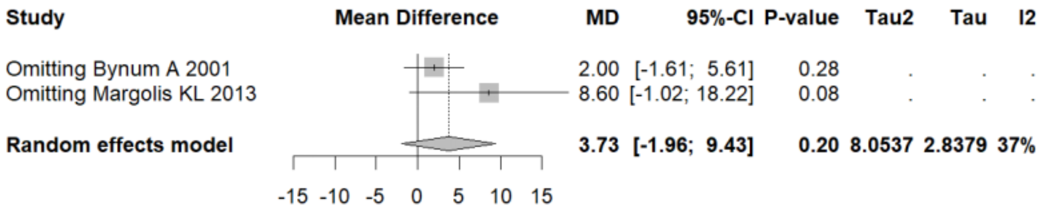

15.3 Adverse events

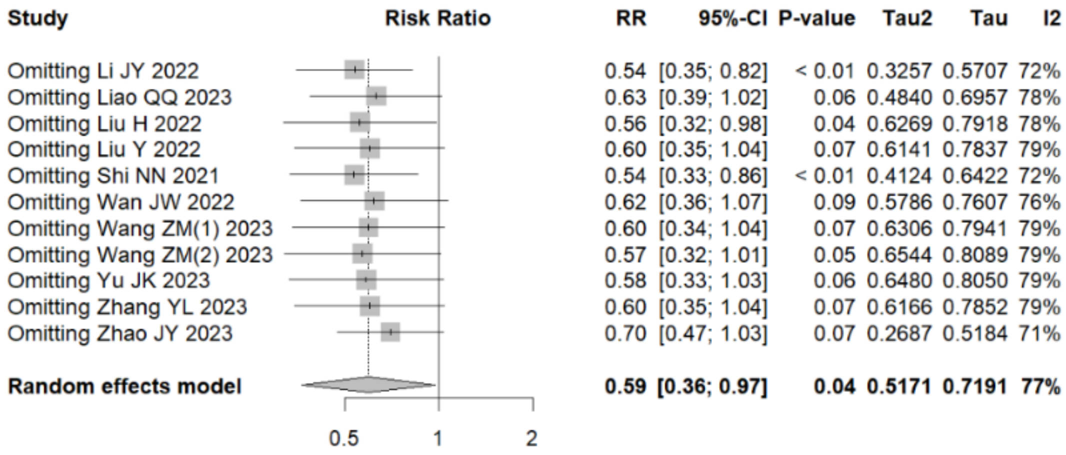

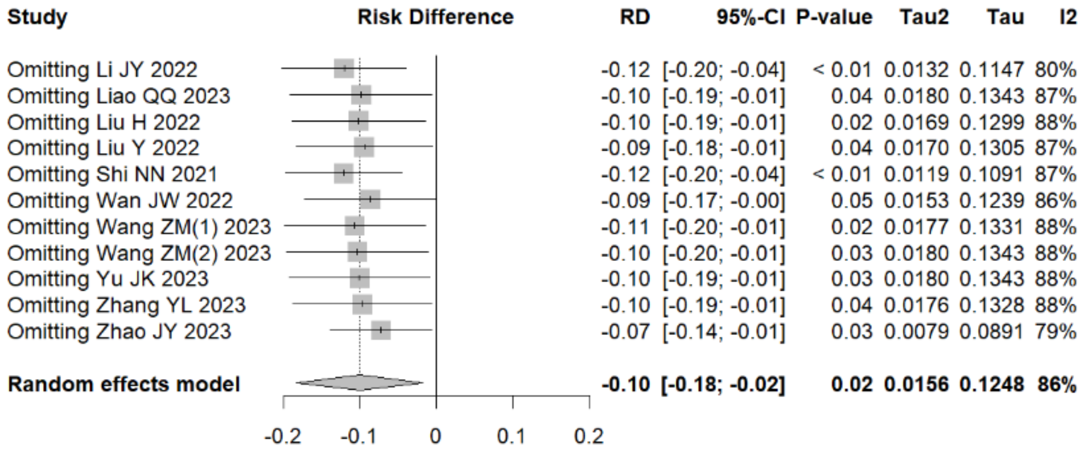

15.4 Diabetes

HbA1c

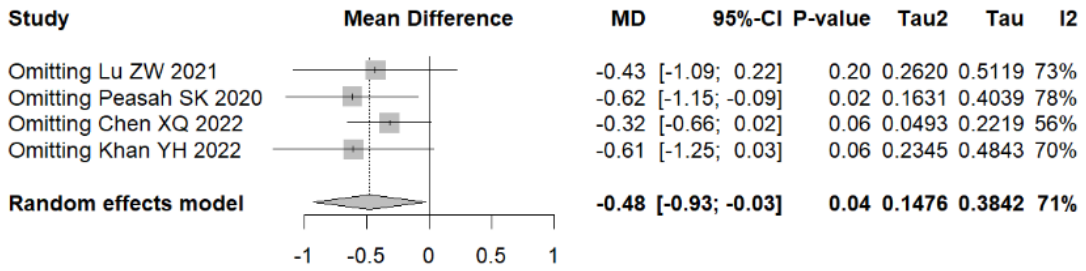

FBG

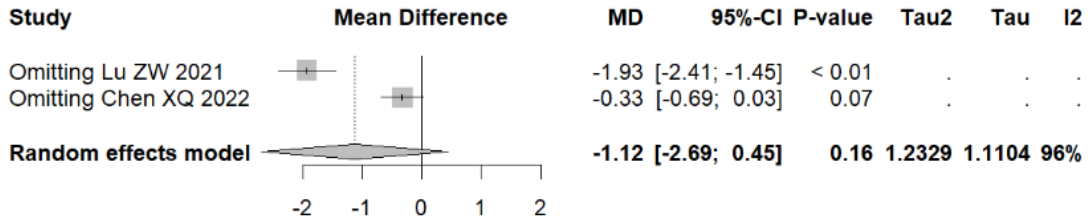

15.5 Hypertension

SBP

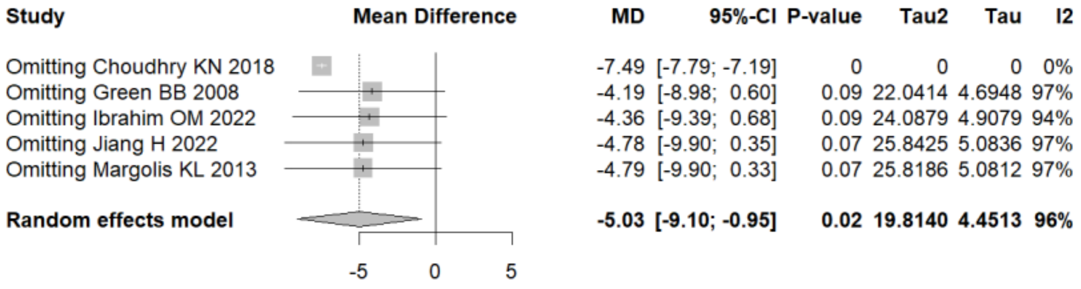

DBP

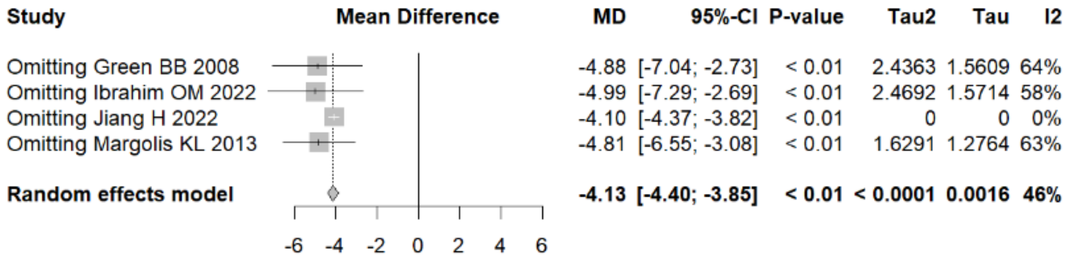

15.6 Anticoagulation

INR (2~3)

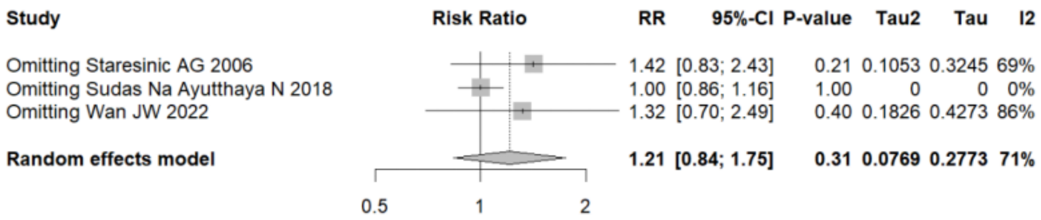

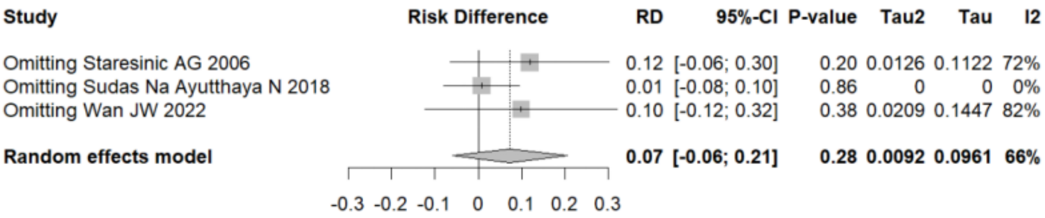

TTR

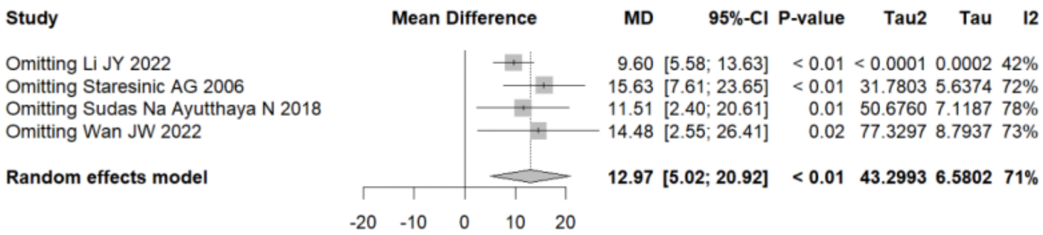

15.7 Other diseases

Respiratory diseases (PEF)

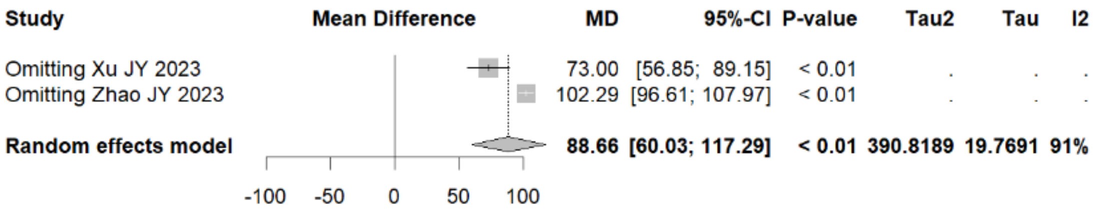

Supplement: Multimedia Appendix 15 [file jmir_v27i1e64073_app15.pdf]
